# Biomarkers in Stress Related Diseases/Disorders: Diagnostic, Prognostic, and Therapeutic Values

**DOI:** 10.3389/fmolb.2019.00091

**Published:** 2019-10-18

**Authors:** Kuldeep Dhama, Shyma K. Latheef, Maryam Dadar, Hari Abdul Samad, Ashok Munjal, Rekha Khandia, Kumaragurubaran Karthik, Ruchi Tiwari, Mohd. Iqbal Yatoo, Prakash Bhatt, Sandip Chakraborty, Karam Pal Singh, Hafiz M. N. Iqbal, Wanpen Chaicumpa, Sunil Kumar Joshi

**Affiliations:** ^1^Division of Pathology, ICAR-Indian Veterinary Research Institute, Bareilly, India; ^2^Razi Vaccine and Serum Research Institute, Agricultural Research, Education and Extension Organization, Karaj, Iran; ^3^Division of Physiology and Climatology, ICAR-Indian Veterinary Research Institute, Bareilly, India; ^4^Department of Genetics, Barkatullah University, Bhopal, India; ^5^Central University Laboratory, Tamil Nadu Veterinary and Animal Sciences University, Chennai, India; ^6^Department of Veterinary Microbiology and Immunology, College of Veterinary Sciences, UP Pandit Deen Dayal Upadhayay Pashu Chikitsa Vigyan Vishwavidyalay Evum Go-Anusandhan Sansthan, Mathura, India; ^7^Division of Veterinary Clinical Complex, Sher-E-Kashmir University of Agricultural Sciences and Technology of Kashmir, Srinagar, India; ^8^Teaching Veterinary Clinical Complex, College of Veterinary and Animal Sciences, Govind Ballabh Pant University of Agriculture and Technology, Pantnagar, India; ^9^Department of Veterinary Microbiology, College of Veterinary Sciences and Animal Husbandry, Agartala, India; ^10^Tecnologico de Monterrey, School of Engineering and Sciences, Monterrey, Mexico; ^11^Department of Parasitology, Faculty of Medicine, Center of Research Excellence on Therapeutic Proteins and Antibody Engineering, Siriraj Hospital, Mahidol University, Bangkok, Thailand; ^12^Division of Hematology, Oncology and Bone Marrow Transplantation, Department of Microbiology & Immunology, Department of Pediatrics, University of Miami School of Medicine, Miami, FL, United States

**Keywords:** biomarkers, stress, diagnosis, prognosis, therapeutic values, acute phase proteins, heat shock proteins, miRNAs

## Abstract

Various internal and external factors negatively affect the homeostatic equilibrium of organisms at the molecular to the whole-body level, inducing the so-called state of stress. Stress affects an organism's welfare status and induces energy-consuming mechanisms to combat the subsequent ill effects; thus, the individual may be immunocompromised, making them vulnerable to pathogens. The information presented here has been extensively reviewed, compiled, and analyzed from authenticated published resources available on Medline, PubMed, PubMed Central, Science Direct, and other scientific databases. Stress levels can be monitored by the quantitative and qualitative measurement of biomarkers. Potential markers of stress include thermal stress markers, such as heat shock proteins (HSPs), innate immune markers, such as Acute Phase Proteins (APPs), oxidative stress markers, and chemical secretions in the saliva and urine. In addition, stress biomarkers also play critical roles in the prognosis of stress-related diseases and disorders, and therapy guidance. Moreover, different components have been identified as potent mediators of cardiovascular, central nervous system, hepatic, and nephrological disorders, which can also be employed to evaluate these conditions precisely, but with stringent validation and specificity. Considerable scientific advances have been made in the detection, quantitation, and application of these biomarkers. The present review describes the current progress of identifying biomarkers, their prognostic, and therapeutic values.

## Introduction

Conceptually, stress has been considered to have a negative connotation in the available literature. However, stress is an inevitable response in all mammals to maintain their homeostasis. Both human and animal health and animal production are hampered severely by different stresses (Martin et al., 2011; Takahashi A. et al., 2018. Strategies to counteract stress in humans and animals often rely on the early detection of stress-induced damage. Various indicators have been identified as potent markers of different biological processes, such as pathogenic or pharmacological responses, and are designated as biomarkers (Griffiths and Moller, 2002; Dadar et al., 2016; Abbas et al., 2017; Prajapati et al., 2017; Selleck et al., 2017; Ewert and Chang, 2018; Tampa et al., 2018). These include normal physiological biomarkers that are within the normal range in healthy subjects. However, a stress marker indicates that an individual is not in physiological comfort and different energy consuming mechanisms are operating inside their bodies to maintain the homeostasis (McEwen, 2015; Marco-Ramell et al., 2016), with the involvement of numerous biomarkers. Hence, a biomarker is a characteristic that can be objectively measured and evaluated as an indicator of a physiological as well as a pathological process or pharmacological response to a therapeutic intervention (Naylor, 2003). According to FDA, an ideal biomarker could be specific for a particular disease and should be able to differentiate between different physiological states, safe and easy to measure, rapid so as to enable faster diagnosis as well as able to give accurate results and consistent between different ethnic groups and genders (Jain, 2010; Sahu et al., 2011). Biomarkers help not only in disease diagnosis but also in tracking progression, regression, and outcome after the intervention. They should be quantified either in the body fluid or externally. Physiological parameters, such as the respiration rate, pulse rate, and core body temperature, are the best-observed markers depicting environmental, social, and psychological stresses (Carboni, 2013). Classical stress markers comprise endocrine changes, especially in the levels of hormones, such as cortisol and epinephrine (Martin et al., 2011; Ewert and Chang, 2018; Takahashi A. et al., 2018). It is the hypothalamic-pituitary-adrenal axis, along with autonomic nervous system, and the immune system that gets sensitized and responds immediately to the peripheral stresses through the commonly known stress biomarkers, such as cortisol, alpha-amylase, pro-inflammatory cytokines (Nater et al., 2013; Ewert and Chang, 2018; Takahashi A. et al., 2018). Deciphering the interaction of different immune cytokines with neuronal circuits of stress is critical to delineate the physiological and psychological stress responses and the prognosis of illness (Godoy et al., 2018). It is proved, experimentally, that hyperthermia and early life stresses in the murine model resulted in deregulation of hypothalamic-pituitary-adrenal (HPA) axis, skewing of hippocampal glucocorticoid receptor mRNA expression and defective neurogenesis indicated by the immature neuron marker doublecortin in an age-dependent manner (Umeoka et al., 2019). Advances in proteomic research have come up with various potential proteins as a panel of biomarkers for diagnosis and therapy related to immunity, blood coagulation, management of oxidative stress, energy metabolism, etc. (Silva-Costa et al., 2019).

From the stress induced physiological and endocrine alterations, disturbances in corresponding functional (e.g., clinical parameters), biochemical (e.g., hormones), metabolic systems become inevitable and hence alterations in metabolic biomarkers (metabolites, enzymes, hormones) also result (Fortunato et al., 2018; Hefnawy et al., 2018). This cascade influences other vital early responding (e.g., cardiovascular, CNS, renal) and late responding (e.g., hepato-biliary, pancreatic) systems. Thus, results in multisystem involvement and hence widespread disturbance in a range of biomarkers peculiar to each system or organ thereof (O'Brien et al., 2017; De Rosa et al., 2018; McGarrah et al., 2018; Nadkarni et al., 2019). The biomarkers concerning cardio-vascular-metabolic function and health include those of vascular function (FMD, BP, AIX), vasculature (cholesterol, HDL, LDL, SAA, sICAM, sVCAM), vascular cytokines (fasting), homocysteine, magnesium (urine), cardiac [(troponins, C-reactive protein, myeloperoxidase (MPO), natriuretic peptides] (Chacko et al., 2017; Dookhun et al., 2018) and corresponding metabolic parameters [(total, HDL, LDL cholesterol (fasting), TG (fasting), glucose, insulin (fasting), HbA1c)]. Similarly, biomarkers for health and function of liver (ketone bodies, central metabolism, ALAT, ASAT, ALP, GGT, CRP, TG, liver IR index, liver IS index), pancreas (disposition index, C-peptide, insulin, glucagon, HOMA-B), kidney (creatinine, Asp, Glu, Orn, urea, albumin), adipose tissue (glycerol, NEFA, and specific FFA, MG, DG, leptin, adiponectin, estimated SCD activity, C16:1 FFA, adipose IR index), gut (fructose, ribulose/xylulose, GIP, GLP-1, indole-3-proprionic acid), brain (secondary messengers, Trp, Tyr, Phe, Met), and muscle (lactate, beta-alanine, muscle IR index, branched chain amino acids and derivatives, 1-methylhistidine, 3-methylhistidine, 4-hydroxyproline, 4-oxoproline) have been enumerated (Chacko et al., 2017; Wopereis et al., 2017; Dookhun et al., 2018; Ho et al., 2018; Karwi et al., 2018; Kyle et al., 2018; Marcato et al., 2018; Pleil et al., 2018). Though these biomarkers constitute the routine health or function(s) detecting biomarkers of these systems, however the persistent alteration under a constant stimulus or etiology results in a disturbance in physio-biological-metabolic homeostatic mechanisms. This causes alteration in endpoint products or by-products of each physiological or metabolic process. Indirectly, the accumulation of these products causes a disturbance in the natural balance of systems and directly persistent stimulus, or etiology can influence normal health and functioning of vital organs (cardiovascular, CNS). All these events starting from disturbance to physiological, endocrine, multiorgan-metabolic to homeostatic mechanisms lead to a state of stress. This initiates a new series of reaction cascade involving oxidative, inflammatory and genomic and proteomic reactions giving origin to particular biomarkers of stress (Dhawan et al., 2014; Alicka and Marycz, 2018; Böbel et al., 2018; Fioranelli et al., 2018; Gómez-Serrano et al., 2018; Ho et al., 2018; Messina et al., 2018; van der Reest et al., 2018; Whongsiri et al., 2018).

These stress-induced reactions are so interlinked that generation of one species (e.g., oxidants, pro-inflammatory cytokines) influences the formation of others (e.g., anti-oxidants, anti-inflammatory cytokines). This, in turn, affects the levels of reactive oxygen species (ROS), and inflammatory mediators (Alicka and Marycz, 2018; Pickering et al., 2018; Saban et al., 2018; Sharma et al., 2018; Yatoo et al., 2019a). This interwoven homeostatic mechanism disturbance results alteration of protective defense mechanisms and result the stress of varying degrees and types. This further aggravates responsive cascade, activating genomic, and proteomic response expressing genes translating to proteins of interest. These all metabolomic, oxidative, inflammatory, genomic or proteomic alterations ultimately serve as biomarkers of stress (Alicka and Marycz, 2018; Nallagangula et al., 2018; Pickering et al., 2018; Sharma et al., 2018; van der Reest et al., 2018; Whongsiri et al., 2018).

Among various forms of stresses, endogenously oxidative and inflammatory stresses are the main generators of various biomarkers that correspond to alteration in different cellular systems they represent (Pickering et al., 2018; Virzì et al., 2018; Gabriela et al., 2019; Yatoo et al., 2019b). There exists a narrow margin of oxygen balance at the cellular level between the production of ROS and the effects of anti-oxidants. During different types of stress, powerful anti-oxidants in mammalian cells, such as glutathione peroxidase and catalase, scavenge these ROS and free radicals (Rahal et al., 2014). Highly reactive unpaired electrons present in ROS and free radicals are unleashed during oxidative stress and can be used as biomarkers at the cellular level (Ho et al., 2013). These free radicals stimulate an array of inflammatory reactions, generating numerous inflammatory mediators that also serve as stress biomarkers (Saban et al., 2018; Sharma et al., 2018; Yatoo et al., 2019b). Immunological stimulation of body defense systems by these alterations or the resulted products further extends the responsive cascade to molecular events including gene expression or protein translation (Yatoo et al., 2018, 2019b). In addition, various proteins get expressed under varying stresses and can be evaluated as biomarkers, e.g., heat shock proteins (HSPs), which are the molecular chaperonins that protect cells from misfolding of denatured proteins during heat-induced stress (Mori et al., 2016) or diseases (Lechner et al., 2018; Tang T. et al., 2018), may also represent useful biomarkers.

Along with the identification of potent biomarkers, the criteria for ideal markers recommend the provision of non-invasive biological samples, such as easily accessible external body secretions. Studies in the past few decades have identified biomarkers that have the potential to revolutionize medical science in terms of diagnosis, prognosis, and therapy (Chowdhury et al., 2013; Selleck et al., 2017; Cesano and Warren, 2018). Biomarkers have been identified for various diseases and disorders; for example, metabolic disorders (Boenzi and Diodato, 2018), cardiovascular disease (Ho et al., 2018), myocardial infarctions (Ge et al., 2018), gynecological diseases (Flores et al., 2018; Liu et al., 2018), neurological disorders (Lashley et al., 2018) and hepatic diseases (Wallace et al., 2016; Raghu et al., 2018). In the present era of high cancer prevalence, sensitive neoplastic biomarkers are a significant research focus, which could aid the early detection and prognosis of neoplastic changes (Tainsky, 2009; Admoni-Elisha et al., 2016; Andersen et al., 2017; Liu et al., 2018). In addition to conventional biomarkers, advances in molecular medicine have identified cell-free nucleic acids, including DNA, mRNA, and microRNAs (miRNAs) as potential markers for several diseases (Lo et al., 2007; Swarup and Rajeswari, 2007; Gilad et al., 2008; Shen et al., 2016; Hibner et al., 2018; Lin et al., 2018). There is an increasing demand for the evaluation of stress to reflect physiological well-being, nutritional status, disease progression, and the immune compromised state. Further, biomarkers can be of diagnostic, prognostic, or therapeutic value (FDA-NIH Biomarker Working Group, 2016). Diagnostic biomarkers help in diagnosing the stress and/or related disease when the prognostic biomarkers are being explored for studying progression or outcome of this stress-disease cascade and predict the likelihood of occurrence of disease. Similarly, therapeutic biomarkers help in monitoring the effect of therapy on stress or disease (Carlomagno et al., 2017; Verber et al., 2019).

Some biomarkers determine the extent of damage and serve as indicators of degradation by stress or disease, such as MDA, isoprostanes, while others, such as anti-oxidant markers reflect a status of body's defense mechanism against stress-induced alterations. Among them, some have dual nature of being both body's normal excretory or metabolic products and anti-oxidant defense, e.g., urates. Some hormones are the normal mediators of the stress process, such as cortisol and adrenaline, while as copeptin or chromogranin A (CgA) prohormones can indirectly determine renal, cardiovascular, or neuroendocrine dysfunction. Enzymes, such as alpha-amylase and lysozyme also serve as biomarkers of stress. Some proteins, such as secretory IgA and heat shock proteins (HSPs) serve as indicators of immunity or resistance mechanism to stress, while as acute phase proteins reflect body response to invading agents. So a broad range of areas concerning these biomarkers needs to be discussed.

Therefore, the present review focuses on the current progress of identifying biomarkers for different stresses in humans and animals, and their prognostic and therapeutic values in stress-mediated diseases and disorders, as well as discussing their futuristic perspectives. It has scope for identification of novel biomarkers with ease of evaluation and accuracy of determination, role in stress and disease, prediction, progression, and monitoring amelioration. The markers discussed include malondialdehyde (lipid peroxidation marker), isoprostanes, enzymatic anti-oxidants, blood urates, cortisol, copeptin, alpha-amylase, secretory IgA, chromogranin A (CgA), lysozyme, microRNAs (miRNAs), heat shock proteins (HSPs), and acute phase proteins. Their utility has been elaborated in various stresses, and related diseases and disorders (Vaishya et al., 2018; van't Erve, 2018; Pulvirenti et al., 2019).

## Types of Biomarkers

Though biomarkers have been classified on various basis including characteristics, application, genetics and molecular biology methods, however biomarkers can be of dual nature or roles and fitting in diverse classifications. As per characteristics, they can be imaging biomarkers or non-imaging biomarkers (Huss, 2015). Imaging biomarkers are applied in identifying or visualizing a lesion or a disease as in computed tomography, positron emission tomography, or magnetic resonance imaging. Non-imaging biomarkers also considered as molecular biomarkers are biochemical type of biomarkers having biophysical properties, hence can be measured in biological samples. They include cellular structures or biophysical components, such as nucleic acid-based biomarkers including gene mutations or polymorphisms and quantitative gene expression analysis, peptides, proteins, lipids metabolites, and other small molecules.

According to the application, they can be classified as diagnostic, prognostic, and therapeutic biomarkers (Drucker and Krapfenbauer, 2013; Huss, 2015). Diagnostic biomarkers are those that help in disease diagnosis or determination. Prognostic biomarkers help in forecasting or likely prediction of disease outcome. Therapeutic biomarkers help in monitoring treatment progress of the disease.

According to genetics and molecular biology methods (Sahu et al., 2011), biomarkers can be categorized into three types, i.e., (1) Type 0, (2) Type 1, and (3) Type 2. Type 0 biomarkers are natural history biomarkers and help in measuring the natural history of the disease and correlate over time with known clinical indicators. Type 1 biomarkers are drug activity biomarkers and indicate the effect of drug intervention. They include efficacy biomarkers which indicate therapeutic effects of a drug, mechanism biomarkers which give information about the mechanism of action of a drug, and toxicity biomarkers that indicate the toxicological effects of a drug. Type 2 biomarkers are the surrogate markers and serve as a substitute for a clinical outcome of a disease. Type 2 also helps to predict the effect of a therapeutic intervention (Jain, 2010).

Another classification, as per Drucker and Krapfenbauer (2013), divides biomarkers into prognostic biomarkers that help in fore-knowing or foreseeing of disease and can tell likely outcome of a disease in an untreated individual. Predictive biomarkers are used to identify patients that can positively respond to a given treatment. Pharmacodynamic biomarkers help in determining the pharmacological effects of a drug. Surrogate endpoint biomarkers have been discussed previously.

As per Mayeux (2004) biomarkers are divided into biomarkers of exposure or antecedent biomarkers that are used in risk prediction and biomarkers of disease that are used in the diagnosis and tracking the progress of a disease.

Despite such classifications, biomarkers have their relevance to each stress mechanism or disease or the organ or system involved. Hence, the description of individual biomarker(s) can be useful in elucidating diagnostic, pathophysiological, and clinical significance.

### Oxidative Stress as Biomarkers

Homeostasis is achieved by the timely maintenance of interactions among the various organ systems, as well as the balance between metabolic processes, their products, and by-products. Various chemical and biological processes elicited within active tissues and cells release oxidative by-products, such as ROS, which include hydrogen peroxides, superoxide anions, reactive chloride ions, and reactive nitrogen species (RNS), such as nitric oxide (Puppel et al., 2015). Normally, cells have several anti-oxidants to counter the damaging effects of oxidative chemicals, and a healthy biological balance should be maintained between ROS and anti-oxidants to prevent oxidative destruction of cells and tissues. Any oxidative imbalance resulting in the accumulation of oxidants will inflict oxidative damage on cells, such as alteration of cellular macromolecules, lethal changes in genetic materials, such as DNA and RNA, an increase in the rate of cell death by programmed- and non-programmed-cell death (apoptosis/pyroptosis/necroptosis/ferroptosis or necrosis), and structural damage to tissues and organs (Sordillo and Aitken, 2009). Accumulation of oxidants can also induce lipid peroxidation and disturbances in physiological adaptation and cellular signaling pathways; which, together, inflict oxidative stress (Yoshikawa and Naito, 2002; Puppel et al., 2015). Recent studies have come up with assessing the levels of and functional interactions between various reactive species interactome (RSI), such as ROS, RNS, etc. in arterial and venous circulation during metabolic and environmental stress. Such redox metabolic approaches were revealed with dynamic pattern of responses and variation in arterio-venous concentration of these metabolic signatures (Cumpstey et al., 2019).

Although oxidants are accumulated in the body primarily endogenously, especially *via* cellular respiration and the electron transport chain, their levels can be augmented from exogenous sources. The major exogenous sources of oxidative attack include radiation (both ionizing and non-ionizing), atmospheric pollutants, biological and chemical toxins, toxic gasses, such as ozone, and oxidizing disinfectants (Eaton, 2006). In addition, foreign microbes invading the body and ingested foods with low nutrient value can lead to the production of tissue/cell-damaging oxidants by disturbing immune responses (Chen et al., 2000; Lykkesfeldt and Svendsen, 2007; Ho et al., 2013). Metabolic disturbances also cause the generation of free radicals (Alicka and Marycz, 2018; Messina et al., 2018). Moreover, strongly indicated oxidative stress biomarkers out of protein oxidation, such as advanced oxidation protein products (AOPP) are also linked with polymorphonuclear neutrophil proliferation and function. This interaction points to the involvement of oxidative stress associated formation of carbonyls and dityrosine residues in uterine inflammations leading to low fertility (Gabai et al., 2019).

Oxidative stress mediated by reactive oxygen and nitrogen species affects vital physiology directly and at the same time, exerts a priming role in the progression of several degenerative conditions and disorders, including cancers, immune disorders, and cardiovascular changes (Lykkesfeldt and Svendsen, 2007; Sordillo and Aitken, 2009; Rahal et al., 2014). Several studies have noted the negative effects of oxidative stress on various pathological processes in animals, including pneumonia and bacterial sepsis in pigs, recurrent airway obstruction in horses, and parturition and lactation induced metabolic disorders in cattle (Basu and Eriksson, 2001; Deaton et al., 2004, 2005; Lauritzen et al., 2005; Castillo et al., 2006). Worldwide, studies in humans and animals indicate the relevance of the timely identification of oxidative stress to ensure the optimum production and health of individuals. Several biomarkers have been identified as cellular oxidative stress indicators in animals. These include the plasma and serum levels of malondialdehyde (MDA), isoprostanes, glutathione (GSH) (L-γ-glutamyl-L-cysteinylglycine), and ROS reduction catalyzing enzymes, such as superoxide dismutase, catalase, glutathione peroxidase, and thioredoxin reductase (Marchitti et al., 2008; Ho et al., 2013; Yatoo et al., 2019b). Both ROS and oxidative stress are very well-related to each other. Imbalances in ROS homeostasis, caused by impairments in anti-oxidant enzymes or non-enzymatic anti-oxidant networks, lead to an increase in oxidative stress. This further causes deleterious oxidation and chemical modification of biomacromolecules, such as lipids, DNA, and proteins. While many ROS are intracellular signaling messengers and most products of oxidative metabolisms are beneficial for normal cellular function, the elevation of ROS levels by light, hyperglycemia, peroxisomes, and certain enzymes causes oxidative stress-sensitive signaling, toxicity, oncogenesis, neurodegenerative diseases, and diabetes (Umeno et al., 2017; Yatoo et al., 2019a). Moreover, reactive oxygen and nitrogen radicals, which are the mediators of oxidative and nitrative stresses, respectively, are being directly linked to systemic metabolic disease, such as diabetes mellitus (Rani and Mythili, 2014; Srinivasan et al., 2018) and associated complications, such as arteriolar sclerosis and nodular glomerulosclerosis, cerebrovascular disease, and amyloid deposition in the pancreas and kidney (Johar and Bernstein, 2017). Hence they have clinical relevance also.

Enzymatic anti-oxidants mediate their beneficial effects *via* the selenocysteine residues in their active sites and have been studied extensively in humans and livestock, particularly in dairy cattle. In addition to anti-oxidant enzymes, non-enzymatic anti-oxidants, such as tocopherols, ascorbic acid, lipoic acid, and carotenoids, also exist, especially in the biological membranes, with vitamin E and α-tocopherols being predominant (Halliwell, 2007). Among the various cellular and tissue systems, red blood cells (RBCs) are uniquely vulnerable to oxidative stress due to the lack of nucleus and mitochondria, inability to synthesize fresh protein along with degradation of detoxifying enzymes, etc. So they are among the first cells to be affected by alterations in the redox status of the body and can be explored for the early detection of pathophysiological alterations of the body in early stages (Pandey and Rizvi, 2011).

Currently, methods are available to evaluate the total anti-oxidant status in animals instead of the individual assessment of each oxidative stress marker. Hence, evaluation of the total anti-oxidant status (TAS) provides critical information concerning the *in vivo* dynamic equilibrium between pro-oxidative and anti-oxidative molecules (Lykkesfeldt and Svendsen, 2007; Ziech et al., 2010; Rani and Mythili, 2014; Yatoo et al., 2019b).

Oxidative stress has been associated with many obesity-related conditions among children, such as cardiovascular disease (Sharma et al., 2018), diabetes mellitus (Pickering et al., 2018), and hypertension (Small et al., 2018). Most of the oxidative stress markers are associated with blood metabolites, such as LDL, cholesterol and other critical biochemical parameters, indicating their crucial influence in lifestyle diseases (Praticò et al., 2004; Patrono et al., 2005; Alicka and Marycz, 2018; Sharma et al., 2018). A prostaglandin-F2α isomer, 8-isoprostane (8-ISO), is created *in vivo* by free radical-catalyzed peroxidation of arachidonic acid. In patients with chronic obstructive pulmonary disease (COPD) and healthy smokers, exhaled 8-ISO is known as an *in vivo* biomarker of lung oxidative stress (Montuschi et al., 2000; Van't Erve et al., 2016). For example, elevated oxidative stress, as indicated by increased 8-ISO levels produced *via* estrogen-related mechanisms, could induce a condition of persistent platelet activation, which promotes the growth and progression of breast cancer through the release of bioactive stored molecules, ultimately contributing to tumor invasiveness (Ferroni et al., 2017). By contrast, in a birth cohort residing in an agricultural area of California, changes in the levels of urinary 8-ISO were associated positively with maternal prenatal urinary levels of phthalate metabolites for 258 participating children at 5, 9, and 14 years of age (Tran et al., 2017).

Protein carbonyl groups are reported as biomarkers of protein oxidation (Dalle-Donne et al., 2003). High levels of protein carbonyl (>C=O) groups have been observed in some diseases, including Alzheimer's disease (AD), rheumatoid arthritis, diabetes, sepsis, chronic renal failure, and respiratory distress syndrome (Pullaiah et al., 2018). In microbial infections (e.g., leptospirosis), induced oxidative stress like the protein carbonyls are of diagnostic value (Fernando et al., 2016). Also, it was reported that urinary 8-hydroxy-2-deoxyguanosine (8-OHdG), an oxidized nucleoside of DNA, is a DNA oxidative stress biomarker and a risk factor for cancer, atherosclerosis, and diabetes (Wu et al., 2004; Kawai et al., 2018). In diabetic patients with hyperglycemia, increased urinary 8-OHdG and leukocyte DNA has been reported, and the urinary 8-OHdG level in diabetes patients has been linked with the severity of diabetic nephropathy and retinopathy (Zhang G. et al., 2018; Zhang L. et al., 2018; Zhang X. G. et al., 2018). Various methods, including high-performance liquid chromatography (HPLC), with and without extraction, and enzyme-linked immunosorbent assays (ELISAs), are proposed to determine 8-OHdG in tissues and urine (Wu et al., 2004; Kawai et al., 2018). Recently, the nanotechnology-based method has been used for determination of 8-OHdG as a biomarker of oxidative stress (Manavalan et al., 2018). Contrastingly, exposure to carcinogens correlates with DNA oxidative damage and most associations of exposures are with urinary 8-OHdG (Franken et al., 2017). Furthermore, 8-OHdG and nuclear factor-kappa B (NF-κB) immunopositivity was reported in brain tissues of rainbow trout exposed to linuron, a herbicide used widely to control grasses and annual broadleaf weeds (Topal et al., 2017). Expression of oxidative stress biomarkers, namely hexanoyl-lysine (HEL), can be used for measurement of lipid peroxidation and 8-OHdG for measurement of DNA oxidation. These biomarkers can also be identified in human tears (Haworth and Chandler, 2017). Oxidation products of linoleic acid, such as hydroperoxides and hydroxides that constitutes hydroxyoctadecadienoic acid (HODE) in biological fluids and tissue samples are the potent lipid peroxidation biomarkers, and their levels will be much higher in conditions like lifestyle-related diseases, such as diabetes and others (Yoshida et al., 2015).

In oxidative stress, a potent biomarker is oxidized low-density lipoprotein (oxLDL) which is measured in relation to certain disease conditions including atherosclerosis (Stocker and Keaney, 2004; Itabe et al., 2018). Plasma is the most common source for measuring oxLDL. Monoclonal antibodies, i.e., 4E6, DLH3, and E06, are the most frequently used to isolate the oxLDL biomarker immunologically. The 4E6 antibody binds to lysine residues on LDL whereas DLH3 as well as E06 recognizes phosphatidylcholine. It is important to note that oxLDL level is high in plasma in patients suffering from cardiovascular diseases or in patients having increased resistance to insulin, diabetes or obesity (Frijhoff et al., 2015; Trpkovic et al., 2015).

Thiobarbituric acid-reactive substances (TBARS) is another biomarker, the level of which can be measured in plasma as well as sera and blood cells, such as RBCs and leukocytes (Cristalli et al., 2012; Moretti et al., 2018) or tissue samples (Yatoo et al., 2016; Moretti et al., 2018). With the help of meta-analysis, the levels of TBARS in Alzheimer's disease (AD) as well as cognitive impairment (mild) have been measured and found to be high in the sera of the patients suffering from AD (Schrag et al., 2013).

Sports sessions produce oxidative stress, and recent studies have found that an increase in ALT level can be employed as a biomarker for athletes to measure the stress level (Mello et al., 2017). Multiple candidate biomarkers for exercise, such as oxidative stress along with brain-derived neurotrophic factor are the main promising components for assessing the anti-depressant effect of exercise, rendering promising adjunct treatment for mood disorders (Gu et al., 2016; Hearing et al., 2016). Oxidative damage of placenta in early gestation can contribute to the progression of pregnancy-associated complications, such as pre-eclampsia, gestational-diabetes mellitus, preterm birth, and intrauterine growth restriction in the later stage of pregnancy. Based on this fact, few of the biomarkers, such as peroxilipids, malondialdehyde, etc. have been suggested to aid in disease diagnosis during early stages in gestation (Cuffe et al., 2017).

Furthermore, decreased concentrations of bilirubin, a significant anti-oxidant, reveal an increase in oxidative stress and have been proposed as a stress biomarker in some epidemiological studies (Vaishnav et al., 2015; Estrada et al., 2016). Similarly, other non-enzymatic natural antioxidants, such as ascorbic acid (vitamin C), alpha tocopherol (vitamin E), glutathione, and uric acid (Bartoli et al., 2018) have been evaluated as oxidative markers (Kawamura and Muraoka, 2018). Another study revealed that hydroxylated polybrominated diphenyl ethers (PBDEs) and their possible metabolites promote oxidative stress in cellular studies (Costa et al., 2014; Yuan et al., 2017). Also, other *in vivo* and *in vitro* studies demonstrated that some PBDEs could induce oxidative stress and inflammation (Fernie et al., 2005; He et al., 2008; Costa et al., 2014). N-3 polyunsaturated fatty acids (PUFAs) can alleviate oxidative stress, as measured by the ratios of late-stage lipid peroxidation markers [malondialdehyde (MDA), 4-hydroxy-2-nonenal (4-HNE), and 8-ISO] to an early-stage marker, lipid hydroperoxide (LPH), which is common in coronary artery disease, and might contribute to depressive symptoms (Mazereeuw et al., 2017). The role of oxidative stress markers in the pathophysiology of asthma has been recognized and reported by Aldakheel et al. (2016), revealing that an elevated level of exhaled hydrogen ions, nitric oxide products, hydrogen peroxide, and 8-isoprostanes in the exhaled breath condensate (EBC) can be reliable markers for asthma and lower airway functions.

**Figure 1:**
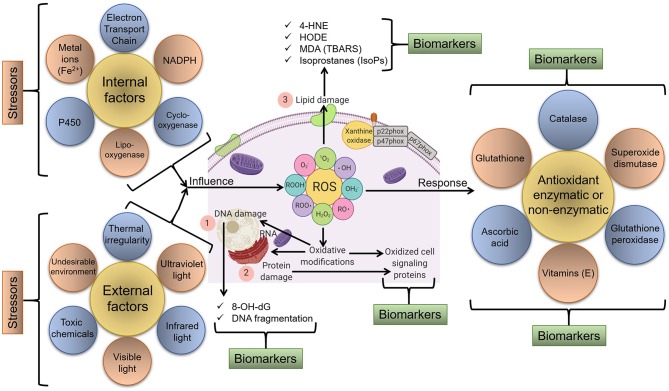
Schematic representation of various endogenous and exogenous factors that act as stressors and lead to the generation of ROS and oxidative stress/modification. In response, various molecular and cellular redox-sensitive processes start that can be tracked as biomarkers of oxidative stress. For instance, major biomarkers include (1) markers of DNA/RNA damage/oxidation, (2) markers of protein damage/oxidation, and (3) markers of lipid damage via the oxidation of membrane components and available lipids, etc.

Figure 1 illustrates a schematic representation of various factors that can act as stressors and lead to the generation of ROS and oxidative stress/modifications that can be tracked as biomarkers of oxidative stress.

Oxidative stress can be physiological to maintain biological processes or pathological to facilitate disease process or stress response (Tan et al., 2018). In either case, alteration in biomarkers can reflect the severity of deviation from normality or degree of damage. A total change in oxidative or anti-oxidative markers through a reliable indicator of alteration but the individual interpretation of each oxidative stress marker will be more accurate for diagnosis and beneficial for understanding the overall process.

### Malondialdehyde (MDA)

One of the most important and extensively studied oxidants is MDA. It is the aldehyde by-product derived from intracellular lipid peroxidation through the action of ROS upon PUFAs. MDA has a longer half-life than ROS; therefore, it can diffuse out to act on both intracellular and extracellular targets, exacerbating oxidative stress in animals (Marchitti et al., 2008; Sordillo and Aitken, 2009; Singh et al., 2013). MDA, along with another aldehyde by-product of lipid peroxidation, trans-4-hydroxy-2-nonenal (HNE), can be detrimental to homeostasis by disturbing the synthesis of essential biomolecules, such as nucleotides and proteins (Barrera et al., 2018). MDA and HNE accelerate the depletion of glutathione, increase proinflammatory cytokines, and activate stellate cells for collagen deposition, which ultimately increases oxidative stress to induce cell/tissue damage (Browning and Horton, 2004). In the field of modern biology to assess oxidative stress, MDA is an extensively utilized biomarker. To predict the pattern of various diseases, such as diabetes, hypertension, cancer, heart failure and atherosclerosis, MDA has been used as a potent biomarker in both *in vivo* as well as *in vitro* studies (Kulkarni et al., 2018). In patients suffering from osteo-arthritis, MDA can be detected in the sections of joint tissue. In both patients suffering from lung cancer as well as glaucoma, the concentration of MDA is high; thereby validating the reliability of MDA assay to find out oxidative stress in relation to the pathology of various diseases (Tiku et al., 2007; Singh Z. et al., 2014). Quantification of the plasma MDA level is evaluated mainly *via* a colorimetric reaction with thiobarbituric acid (TBA) (Meagher and FitzGerald, 2000). Novel antibody-based tests are possible using standard ELISA kits validated against high-performance liquid chromatography (HPLC), which have produced reliable and specific results (Bevan et al., 2003). Recently, a non-invasive method of quantification of MDA biomarker in human exhaled breath condensate using self-assembled organic-inorganic nanohybrid was used, and it has shown promise for diagnosing lung diseases being appropriate, reliable, inexpensive, fast, and user-friendly diagnostic tool (Jafari et al., 2019).

Investigations in dairy cattle indicated that the highest levels of plasma MDA reflected metabolic disturbances, especially around parturition and the early stages of lactation (Turk et al., 2004; Castillo et al., 2005). MDA and total anti-oxidant status (TAS) values are suggested as effective indicators for the oxidant-antioxidant balance and could be employed to generate complementary measures of animal homeostasis (Castillo et al., 2006; Ho et al., 2013). HPLC with diode-array detection (HPLC-DAD) was revealed as a selective, reproducible, and sensitive method to measure MDA in goat plasma as an oxidative stress biomarker (Yonny et al., 2016). In addition, gas chromatography coupled with tandem mass spectrometry (GC/MS/MS) is a useful method in long-term clinical studies of circulating MDA as a biomarker of lipid peroxidation, and its relevance to F2-isoprostane 15(*S*)-8-*iso*-prostaglandin F2α and nitric oxide (NO) has been demonstrated (Tsikas et al., 2016). Another study reported that GC-electron-capture negative ion chemical ionization (ECNICI)-MS measurement of nitrite and malondialdehyde in human urine is important as a surrogate internal standard for MDA (Hanff et al., 2016). A recent study demonstrated malondialdehyde-modified low-density lipoprotein (MDA-LDL) to be a good candidate for predicting the endovascular therapy outcome in patients affected with peripheral artery disease (Takamura et al., 2017). Acute stroke can be identified using levels of MDA (Liu Z. et al., 2017). Recently, MDA has been evaluated as an oxidative stress diagnostic biomarker in diabetes (Yatoo et al., 2016; Ma et al., 2018), ketosis, ovarian cyst, mastitis and lameness (Kapusta et al., 2018). Its elevated concentration in milk and meat has shown deleterious effects on milk (Yatoo et al., 2015; Kapusta et al., 2018), and meat (Cimmino et al., 2018) quality and hence can serve as a quality biomarker in foods.

As MDA is a lipid peroxidation product and determines the extent of damage of biological membranes, it can suitably be evaluated as a biomarker for degradative processes/stresses or diseases.

### Isoprostanes

These are prostaglandin-like compounds released from the peroxidation of arachidonic acid, independently of the normal cyclo-oxygenase pathway, which are subsequently released for circulation by phospholipases (Griffiths and Moller, 2002; Stafforini et al., 2006; Czerska et al., 2016). The presence of isoprostanes, especially F2-isoprostane (F2-Isops), can be quantified from various biological sources, such as blood (Siti Hajar et al., 2018), urine (Roy et al., 2015), cerebrospinal fluid (Finno et al., 2018), or other fluids or tissues (Annelies et al., 2018; Finno et al., 2018; Jadoon and Malik, 2018). F2-Isops, being the most stable among the isoprostanes, is the most potent isoprostane biomarker and its level can reflect the oxidative status of vital organs, such as liver or kidneys (Morrow, 2005; Musiek et al., 2005). In both humans and animals, increased plasma and urine concentrations of F2-Isops correlate significantly with oxidative stress, revealing its potency as a stress marker (Fam and Morrow, 2003; Van't Erve et al., 2016). F2-Isops can be quantified using gas/liquid chromatography associated with mass spectrometry, immunological assays, including ELISA/radioimmunoassays (RIAs), and using commercial assay kits (Musiek et al., 2005; Smith et al., 2011). In addition, elevated levels of the oxidative stress biomarker 8-iso-prostaglandin F2α (8-iso-PGF2α) in wastewater is associated with tobacco use and represents a powerful wastewater biomarker to evaluate community public health (Ryu et al., 2016). Interestingly, it is reported that long-term supplementation with vitamin E reduces oxidative stress in smokers, which is determined by 8-iso-PGF2α detection (Guertin et al., 2016). Isoprostanes have been evaluated as biomarkers in equine neuroaxonal dystrophy (Finno et al., 2018), Creutzfeldt-Jakob disease, Huntington's disease, Alzheimer's disease, multiple sclerosis (Jadoon and Malik, 2018), Attention-Deficit/Hyperactivity Disorder (ADHD) (Annelies et al., 2018), and in passive smokers (Siti Hajar et al., 2018).

Some isoprostanes have found clinical applications in obesity, ischemia-reperfusion injury, the central nervous system, cancer, and genetic disorders (Milne et al., 2015). CSF isoprostane levels have been beneficial in evaluating oxidative stress in multiple sclerosis in humans (Mir et al., 2014) whereas plasma or milk isoprostane levels were diagnostic for oxidative stress in lactating dairy cows (Vernunft et al., 2014).

### Enzymatic Anti-oxidants

Most potent anti-oxidant actions are mediated by enzymes, especially superoxide dismutase, glutathione peroxidase, and catalase, which mediate the direct reduction of ROS (Carocho et al., 2018). Glutathione peroxidase is a selenoenzyme that can reduce large quantities of hydroperoxide radicals in association with thiols like glutathione. The level of serum glutathione peroxidase is an excellent measure of the oxidative status of an individual and is most often employed in diagnostics (Gheita and Kenawy, 2014). Superoxide dismutase (SOD) and catalases reduce ROS directly into metabolic water and oxygen molecules, thereby preventing oxidative damage to tissues. Thioredoxin reductase is another selenoprotein-based antioxidant enzyme that has a critical role in reducing ROS into the less reactive water and alcohol, preventing their oxidant action (Hara, 2001; Grignard et al., 2005; Trigona et al., 2006). At present various reliable commercial assay kits are available to assess these biomarkers effectively (Blankenberg et al., 2003). Fluorometric assays for GSH/GSSG ratio (GSH/GSSG Ratio Assay Kit) and Total Antioxidant Capacity (Total Anti-Oxidant Capacity Assay Kit) from Abnova USA, Abcam USA, Sigma-Aldrich USA, Cayman Chemical USA or Bioassay Systems USA, Immunoassay for ArborAssays USA and Cell Biolabs USA, spectrophotometric method for aconitase (BIOXYTECH® Aconitase-340 Assay) are available. These have been used in numerous oxidative stress-related studies (Ijomone et al., 2014; Chauhan et al., 2017; Zhang Y. et al., 2017; Dada et al., 2018; Peng K. T. et al., 2018). They have been used in clinical applications, e.g., evaluating total anti-oxidant status in diabetic patients (Rani and Mythili, 2014).

Myeloperoxidase is a heme-based enzyme from proinflammatory cells that generate ROS, especially during phagocytosis in response to microbial attack, resulting in modification of biomolecules if not regulated. Spectrometric evaluation of its plasma concentration is a reliable method to assess the oxidative status and subsequent damage *in vivo* (Nicholls and Hazen, 2005). Myeloperoxidase is released into circulation, and its level in serum has been suggested as a useful measure of atherosclerotic plaque vulnerability in cardiac patients (Sugiyama et al., 2004). An elevated myeloperoxidase concentration is documented in various studies of coronary artery diseases, indicating the prognostic significance of myeloperoxidase in these conditions (Sugiyama et al., 2001; Zhang et al., 2001; Tsimikas, 2006). Myeloperoxidase has found clinical applications in rheumatoid arthritis, cardiovascular diseases, liver diseases, diabetes, and cancer (Coculescu et al., 2018; Khan et al., 2018).

A crucial role is played by oxidative stress in periodontitis. The level of SOD is found to be high in periodontitis. Periodontal ligament possesses this enzyme; thereby neutralizing the activity of reactive oxygen species (ROS). The SOD is introduced due to the release of superoxide stimulated by polysaccharide of bacteria. Recently clinical significance of SOD in gastric cancers has been summarized (Li et al., 2019). In chronic cases of periodontitis, the enzyme glutathione peroxidase shows variation in concentration. There may be an increase or decrease in the level of this enzyme (Aziz et al., 2012; Jeeva et al., 2015). In patients suffering from oral cancer, there is a reduction in the activity of catalase enzyme which is attributed to high amount of superoxide anion or reduction in enzymatic scavenging activity (Patel et al., 2009).

Glutathione reductase is another enzymatic anti-oxidant that catalyzes the reduction of glutathione disulfide (GSSG) to the sulfhydryl form glutathione (GSH) (Maciejczyk et al., 2018a). The levels of oxidized glutathione (GSSH) and reduced glutathione (GSH) predict oxidative stress and hence the ratio of GSH/GSSG is used as an oxidative index (Zitka et al., 2012; Giustarini et al., 2016). However, there are concerns regarding the use of this index as a measure of oxidative stress (Sentellas et al., 2014). Still, there are numerous efficient methods of expressing this index (Sentellas et al., 2014). Some prooxidant enzymes (xanthine oxidase and NADPH oxidase) that help in generation of ROS and aggravate oxidative stress, can also serve as diagnostic biomarkers in oxidative stress to evaluate severity (Maciejczyk et al., 2018b; Simioni et al., 2018). In the case of systemic oxidative stress, tissues respond diversely with respect to glutathione concentration. Barry-Heffernan et al. (2019) have reported that the GSH levels in erythrocyte and plasma are not correlated to that in liver biopsy tissues of dogs, which are clinically indicated for liver biopsy, suggesting that the GSH levels in circulatory system may not be ideal for assessing the redox status of the liver.

### Blood Urates

Urates are the major end products of purine metabolism in mammals and have inherent anti-oxidant action. Blood plasma and cerebrospinal fluid (CSF) urate concentrations are associated with cellular models of neurodegeneration, which might be due to their capacity to reduce oxidative stress, mitochondrial damage, and cellular apoptosis (Guerreiro et al., 2009). Peroxyl (ROO^•^) and hydroxyl (OH^•^) radicals, which are capable of inducing DNA damage in cells, are inhibited powerfully by the anti-oxidant potential of urates. Urates' shielding action has also been linked to its capacity to form complexes with metal ions in cells under attack by oxidative damage. Unabated elevation of the ROS and hemoprotein/H_2_O_2_ systems in cells causes oxidation of urates to inactive forms, reducing their potency and efficacy. Laboratory-based epidemiological and clinical investigations in neurological diseases, especially Parkinson's diseases, revealed a negative correlation between blood and CSF urate concentrations and disease progression (Cipriani et al., 2010). Biochemical evidence indicated that the urate level is a promising biomarker to assess the incidence, diagnosis, and therapeutic prognosis of various neurodegenerative conditions, and suggested the possible therapeutic efficacy of endogenous urate molecules. Notably, under normal conditions, the kidneys continuously excrete uric acid out of the body, and the presence of circulating uric acids in the body is an important determinant of renal impairment (Stamp et al., 2011). In addition to being the routine biomarkers of kidney (Klein et al., 2018; Lu et al., 2018) and liver diseases (Drolz et al., 2018), urates or its related products are also proving to have diagnostic value in other important diseases, such as Parkinson's disease (Cova and Priori, 2018), diabetes (Xie et al., 2018), cardiovascular diseases (Murata et al., 2018), obstructive sleep apnea (Fleming et al., 2018), and bone inflammation (Beyazit and Pek, 2018). Urates or urea derivatives have also shown applicable values in clinical diagnosis of renal diseases (Yang et al., 2018), gastrointestinal and hepatic diseases (Siddiqui et al., 2016), using advanced technologies like self-powered implantable electronic-skin and non-invasive breath test, respectively.

### Cortisol

The primary mediator of the physiological changes during stress is the neuroendocrine axis. In this process, the hypothalamic-pituitary-adrenocortical (HPA) system regulates the secretion of cortisol, and the sympathetic adrenomedullary (SAM) system regulates catecholamine secretion. Significant correlations have been observed between salivary cortisol and blood cortisol concentrations, especially during stress. In response to various stresses, the HPA system is activated, which induces the secretion of cortisol into the blood, and in this regard, salivary cortisol can be a reliable estimate of stress-induced HPA activity. Salivary cortisol mostly exists in the unbound (free) form, accounting for ~70% of the total unbound cortisol in the body (Jung et al., 2014). This unbound cortisol, because of its low molecular weight and liposolubility, diffuses through the acinar cells of the salivary gland and is secreted into the saliva (Ivković et al., 2015).

The urinary cortisol concentration cannot be exactly correlated with the blood concentration because of renal tubular reabsorption and secretion; therefore, salivary cortisol represents a good alternative and has become the most frequently studied stress biomarker in salivary samples (Hellhammer et al., 2009). Studies on stress-related depressive disorders revealed that such disorders could be diagnosed through cortisol levels (Islam et al., 2018; Xu Y. Y. et al., 2018). Another study revealed that patients with the lowest cortisol concentrations showed the least improvement in agoraphobic avoidance after psychotherapy (Wichmann et al., 2017), demonstrating an inverse association of the cortisol stress response with agoraphobic avoidance after psychotherapy. Also, in 198 *Rhesus macaques* (89 male) with demonstrated extensive alopecia (>30% hair loss), alopecia, hair loss, and hair cortisol concentrations were associated with elevated chronic cortisol concentrations (Novak et al., 2017). Furthermore, major depression is related to long-term attenuation of cortisol secretion (Steudte-Schmiedgen et al., 2017). Recently, a study was conducted to know the diagnostic value of oxidative stress markers, namely cortisol and α-amylase, in saliva to identify preterm birth in women. Results showed that increased cortisol level during pregnancy could be used as a tool to predict preterm birth (García-Blanco et al., 2017a).

Another way of monitoring stress is the assessment of cortisol from hair samples. This can be a complementary mode of analyzing systemic cortisol over longer periods in comparison to the cortisol concentration in saliva and urine samples which reveal only real-time data. This method finds application in cortisol quantification in chronic inflammations as well as acute conditions like myocardial infarctions (Russell et al., 2012). Stress hormones, like glucocorticoids along with cytokines, act as master homeostatic regulators in circulation, which mediate several conditions like post-traumatic stress disorder (PTSD) along with key vital pathways both peripherally and centrally, and represent promising functional biomarkers of stress responses in human as well as animal subjects; thus can be targeted for developing novel therapeutics (Michopoulos et al., 2015; Daskalakis et al., 2016). Though novel biomarkers are being explored for stress, however, still various recent studies have focused on cortisol of various origins (Egawa et al., 2018; Suh, 2018). Oxidative injury by other oxidants like MDA also causes the release of cortisol and hence both in the association can serve as diagnostic oxidative markers in diseases (Islam et al., 2018; Xu Y. Y. et al., 2018).

Evaluations of novel cortisol in hair or sweat has shown diagnostic value in various forms of stress or diseases, and has found clinical applications in Cushing syndrome (particularly Cyclical Cushing), Adrenal insufficiency (including Addison's disease), therapy monitoring, cardiovascular disease, stress, and mental illness (Meyer and Novak, 2012; Jia et al., 2016; Greff et al., 2019).

### Copeptin

Copeptin is a polypeptide derived from the hypothalamo-pituitary axis-system, as a pre-prohormone along with vasopressin and neurophysin II. This C-terminal derivate of the arginine vasopressin mainly regulates water and electrolyte balance, hence has a diagnostic role in cardio-renal dysfunction (Tan and Sethi, 2014). Even though vasopressin is a major hypothalamic stress hormone, application of circulating vasopressin as a stress biomarker is challenging due to its pulsatile release, instability in plasma and rapid clearance (Morgenthaler et al., 2008). Copeptin, being a co-component of pre-pro-hormone and released as a more stable protein in an equimolar ratio to vasopressin, can be finely employed for assessing the individual stress level in comparison to cortisol (Katan and Christ-Crain, 2010). Clinically the prognostic accuracy of copeptin in acute illness, such as sepsis, pneumonia, lower respiratory tract infections, stroke, has been analyzed successfully (Katan and Christ-Crain, 2010). Because of the molecular stability, easier and faster test procedures and results, copeptin are used as a replacement along with cardiac troponins for faster diagnosis of myocardial infarction. Heart failure (HF) can be predicted well by using copeptin as a biomarker in combination with brain-type natriuretic peptide (BNP). There is a rise in the levels of copeptin in cardiovascular shock (Kristyagita and Siswanto, 2015). In recent times though copeptin is diagnostic biomarker for kidney (Tasneem et al., 2018) and heart (Berezin, 2018; Xu L. et al., 2018) related ailments mainly, but in future has good scope for evaluation of other systems or diseases as these systems are interdependent and affect one another, e.g., cardio-pulmonary or hepato-renal disorders. It has found clinical applications in acute myocardial infarction (Ay et al., 2017; Aydin et al., 2019), polycystic kidney disease (Tasneem et al., 2018), insulin resistance and metabolic syndrome (Saleem et al., 2009).

### Alpha-Amylase

Although the stress-induced sympathetic adrenomedullary system regulates catecholamine secretion, the salivary enzyme α-amylase has been estimated as a marker for sympathetic stimuli (Rai and Kaur, 2011). In normal conditions, the level of this adrenalin-induced enzyme is lowest in the early morning hours and highest in the late afternoon (Takai et al., 2004). In response to stress, the α-amylase concentration increases drastically, indicating that this can be considered as a potent salivary biomarker of stress (O'Donnell et al., 2009; Strahler et al., 2010). Due to the stress (acute) caused during venipuncture, the concentration of α-amylase in saliva increases (Koh et al., 2014). It was suggested that the α-amylase level in saliva could reflect nervous system activity exercise responses in elite male wheelchair athletes, with or without cervical spinal cord injury (Leicht et al., 2017). In recent findings, salivary amylase has been found to be diagnostic in oral disease (Lorenzo-Pouso et al., 2018), renal diseases (Maciejczyk et al., 2018b), cardiovascular and psychological diseases (Mishra, 2017).

Salivary amylase has been used as biomarkers for clinical evaluation of stress in pigs (Escribano et al., 2019), acute abdominal disease in horses (Contreras-Aguilar et al., 2019), neurobehavioral activity (Pajcin et al., 2019) and radiation exposure in humans (Balog et al., 2019).

### Secretory IgA

Various studies have shown interactions between the neuroendocrine system and the immune system, and this also occurs in stress conditions. The induction of stress hormones, especially cortisol, harms immunoglobulin secretion, thereby decreasing the concentration of immunoglobulins in body fluids (Mocci and Bullitta, 2006; Lee et al., 2010). Furthermore, higher salivary IgA, as well as decreased anxiety, were linked with fewer potentially pathogenic oral bacteria and enhanced oral immunity (Lamb et al., 2017). IgA has a diagnostic role in gastrointestinal diseases (Siddiqui et al., 2017), stress-immunity link diseases (Staley et al., 2018), maintenance of the intestinal epithelial barrier, gut health and microbiota regulation (Donaldson et al., 2018; Ducatelle et al., 2018), besides playing role in gut nutrition and immunity (Celi et al., 2018), Hence, IgA is considered as a non-invasive biomarker of gastrointestinal functionality, microbiota, health and immunity (Celi et al., 2018; Ducatelle et al., 2018).

### Chromogranin A (CgA)

CgA is an acidic protein prohormone that is present in the secretory granules of different neuroendocrine tissues, and has been recognized as a marker of mental stress (Yamakoshi et al., 2009). It is stored primarily in adrenal gland vesicles and is released into the circulation, along with catecholamines, *via* exocytosis (Ivković et al., 2015). In normal subjects, higher levels of CgA are observed during the night, and lower levels are seen in the morning (Giampaolo et al., 2002); however, significantly higher concentrations of CgA were observed in saliva samples collected immediately after exposure to various stresses (Ng et al., 2003; Takatsuji et al., 2008). CgA was also proposed as an important biomarker in diabetes (Broedbaek and Hilsted, 2016). Recent studies evaluated CgA as a valuable biomarker in various stressful diseases including neuroendocrine tumors (Di Giacinto et al., 2018), cardiovascular disorders (Ottesen et al., 2017; Mahata et al., 2018), atopic dermatitis (Cai et al., 2018), ulcerative colitis (Magnusson et al., 2018) and diabetes mellitus (Herold et al., 2018).

Serum CgA is considered as the diagnostic biomarker for gastroenteropancreatic neuroendocrine neoplasms and has been utilized in clinical applications (Pulvirenti et al., 2019; Zhang et al., 2019). However, it has not been found effective as a biomarker for diagnosis or management of bronchopulmonary neuroendocrine tumors/neoplasms (Matar et al., 2019). Also, in pancreatic neuroendocrine tumors, CgA as a clinical biomarker has found limited role. Salivary CgA could be used as a potential biomarker in animal production systems to monitor the severity of social stress and behavioral aggression. Chromogranin A is highly correlated with the skin lesions due to fighting in weaning piglets as it is an indicator of activation of sympathetic adrenomedullary stress pathway (Escribano et al., 2019).

### Lysozyme

Lysozyme is a prominent anti-bacterial peptide in the external secretory fluids of humans and animals. This cationic protein shows potent bactericidal actions by hydrolyzing bacterial cell walls (peptidoglycan), especially those of Gram-positive bacteria. Studies have reported that there is a negative correlation between lysozyme concentration and stress exposure, which is in accordance with increased susceptibility to bacterial invasion during stress (Yang et al., 2002; Allgrove et al., 2008). Another study revealed that lysozyme is related to circulating RNA, extracellular vesicles, and chronic stress (Abey et al., 2017). Currently lysozyme as diagnostic biomarker has found applications in chronic stress (Abey et al., 2016), cancer metastasis (Brzozowski et al., 2018), cardiovascular markers, psychological research (Mishra, 2017), oral diseases (Lorenzo-Pouso et al., 2018), diabetes (Maciejczyk et al., 2017), wound healing (Abey et al., 2016), infectious diseases (Ghosh et al., 2018; Stjärne Aspelund et al., 2018), Graves' disease and orbitopathy (Zhang L. et al., 2018). Serum lysozyme has been used as a clinical biomarker for diagnosing sarcoidosis with high specificity (Ramos-Casals et al., 2019). Similarly, plasma lysozyme has been used as a putative biomarker of atherosclerosis (Abdul-Salam et al., 2010).

Conventional biomarkers generally are weak in their sensitivity, specificity, and poor reflectors of the complex interactions underpinning the cellular and molecular changes during stress related disorder and diseases. Complex biological networks delineated through computational algorithms can forecast impending molecular alterations, which may further results in stress disorder or clinical diseases. In this regard, stress response or a clinical illness may be perceived very early through probing the emergent skewing of molecular pathways. At the same context conventional serum, cellular and molecular markers may take enough time to reflect its detectable level in the respective diagnostic fluid, so that computational algorithms tailored for mining the omics data trespassed the many conventional approaches.

## MicroRNAs (miRNAs)

MicroRNAs, short sequences of RNA (~22 nucleotides), are a class of small non-coding RNA segments that are regulated and transcribed like protein-coding genes (Bartel, 2000; Trzybulska et al., 2018). MicroRNAs regulate gene expression post-transcriptionally and influence normal biological processes, as well as various pathological conditions (Gilad et al., 2008). They are also present in cell-free body fluids like serum, suggesting their utility as non-invasive clinical biomarkers for prediabetes, diabetes, and related complications (Vaishya et al., 2018). MicroRNAs in serum was demonstrated to be stable and are sufficiently robust to serve as practicable clinical biomarkers to differentiate the patients with autoimmune disease from healthy individuals (Jin et al., 2018). Easily accessible biomarkers to evaluate and predict pregnancy complications are required urgently; serum levels of miRNAs reflecting critical physiological conditions, such as pregnancy and its associated complications, could be exploited as useful markers, suggesting their clinical utility to determine pregnancy stages and related abnormalities (Liang et al., 2007). Many miRNAs are present in maternal serum and their level increase with gestational age. Recently, distinctive expression of miRNAs in the placenta in association with pre-eclampsia was observed, which highlighted the possibility that serum levels of particular miRNAs might serve as future diagnostic biomarkers for pre-eclampsia (Walker, 2000; Pineles et al., 2007). Further detailed investigations on miRNAs revealed the potential of cell-free miRNAs in body fluids to serve as practical and reliable molecular markers to assess diverse physiological and pathological conditions. Among these conditions, the possible critical roles of miRNAs in tumor diagnosis, disease progression, and prognosis have received increased research attention (Yan et al., 2016). Accumulating data suggest the application of abnormally expressed miRNAs in blood and serum as promising candidates to predict hepatocellular carcinoma (Jiang et al., 2015; Shen et al., 2016). Circulating miRNAs in blood could serve as novel diagnostic markers for various disease conditions owing to their evolutionary conservation and stability. The potential of serum miRNAs as non-invasive biomarkers for assessing the progression of subarachnoid hemorrhage has been recently explored by Lai et al. (2017). Studies are being demanded in elucidating the critical role of circulatory miRNAs as coagulation and thrombosis biomarker in order to find their clinical application in predicting complications like stroke (Vijayan and Reddy, 2016). Their usage as potential biomarkers in equine medicine has been recently reviewed by van der Kolk et al. (2015). MicroRNAs might be exploited as novel diagnostic markers for myopathies, recurrent exertional rhabdomyolysis, and osteochondrosis. MicroRNAs in blood could also be important in glucose metabolism pathway of the equine. Further investigations and validations are required for these novel molecular markers, particularly to determine better reliable body fluid miRNA profiles to exploit their clinical utilization, thereby paving the way for their wider application in the future.

Currently, miRNAs are being studied as potential diagnostic biomarkers in both epidemiological (He et al., 2018) and clinical studies (Pogribny, 2018). They are proving helpful in all fields of medicine, be it for elucidating disease associations (He et al., 2018), etiology (Liguori et al., 2018), diagnosis (Wang H. et al., 2018; Zhou Q. et al., 2018), typing (Pérez-Sánchez et al., 2018), therapeutics (Roy et al., 2018; Zhou, S. S. et al., 2018), progression (Clark et al., 2018), perioperative medicine (Kreth et al., 2018) and much more. In diagnosis, they have been found to possess great applications. They are being used for diagnosis of cancers (Wang H. et al., 2018; Zhou Q. et al., 2018), cardiovascular diseases (Pérez-Sánchez et al., 2018; Zhou, S. S. et al., 2018), hepatic diseases (Schueller et al., 2018), renal diseases (Shaffi et al., 2018), sporadic amyotrophic lateral sclerosis (Liguori et al., 2018), Parkinson's disease (Roser et al., 2018), etc.

## Heat Shock Proteins (HSPs)

Heat shock proteins (HSPs) are molecular chaperones with multiple physiological roles. HSPs comprise highly conserved protein families across different species of animals. The HSP family comprises various critical proteins, such as HSP60, HSP70, and HSP90, among which HSP70 is the most prominent, with significant effects on diverse biological systems and therapeutic potential (Shrestha et al., 2016; Khandia et al., 2017). Various HSPs are induced in response to short-term stress, such as thermal stress, osmotic stress, heavy metal toxicity, and ecological stress from pollutants (Hecker and McGarvey, 2011). Environmental stress acts as predisposing factors for the synthesis and secretion of various heat shock proteins at greater concentration. Such stresses include infection and inflammation, exercise, the cell being exposed to various toxic substances, dearth of water, etc. This is the reason why these proteins are also termed as stress proteins (Santoro, 2000). The mechanisms of activation of heat shock factor by heat shock have been well-described in bacteria. There is no unfolding of outer membrane proteins (OMPs) during stress; thereby insertion of these proteins occurs inappropriately in the outer membrane; ultimately accumulation occurs between the inner cytoplasmic membrane and outer membrane (periplasmic space). A protease of the inner membrane detects these OMPs and the signal is passed to a transcription factor sigmaE (Walsh et al., 2003). Upregulation of certain heat shock proteins of bacteria occurs through RNA thermometers, such as HSP90 cis-regulatory element (Narberhaus, 2010). Post-transcription and translation studies revealed that the expression of HSPs increases dramatically in response to other stresses and degenerative conditions, such as hypoxia-induced tissue injuries, ischemia, and CNS degeneration (Li et al., 2004; Mariucci et al., 2007). These HSPs ultimately serve as the endogenous mediators that initiate intracellular cascades to provide cellular protection from the stresses mentioned above. HSP70 was first reported in the fruit fly, *Drosophila melanogaster*, where it was found to have a half-life of 2 h, after which its activity decreases rapidly (Li and Duncan, 1995). Continuous secretion of HSP70 is induced by ongoing exposure to thermal, osmotic, hypoxic, or toxic stresses. Besides, the co-localization of HSP70 with early indicators of cellular stress, such as the cytokines c-fos and c-jun, has been observed; however, the HSP response is specific to the type of stress and cellular factors. HSPs are governed tightly by cellular regulatory mechanisms, such that those tissues with thermal tolerance can restrict its responses to stresses by eliciting feedback signals that regulate both transcription and translation (Van Eden et al., 2010). Certain non-classical secretory pathways also exist for the active secretion of HSP70, especially those proteins concerned with the stress response in lipid rafts (Multhoff, 2007). This demonstrated the presence of HSP70 in the cellular membranes of tumor–affected cells and by its association with lipid raft receptor complexes (Bausero et al., 2005; Mambula et al., 2007). HSPs, especially HSP70, also induce anti-inflammatory responses, in particular through regulatory T lymphocytes (Tregs) (Mariucci et al., 2007). Role of HSP70 as a biomarker in monitoring environmental diseases, such as mycotoxicoses has been explored and reported to be of potential application (El Golli-Bennour and Bacha, 2011).

**Figure 2:**
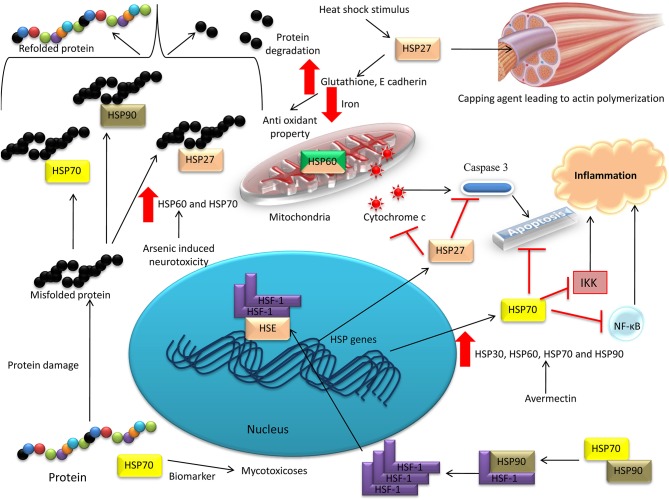
Heat shock proteins as stress biomarkers. HSP70 has been reported as a biomarker in monitoring environmental diseases, such as mycotoxicoses. HSP27 was characterized as a potent marker for thermal stress, facilitating the effective re-orientation of misfolded proteins. HSP27 inhibits the intrinsic and mitochondrial apoptotic pathways *via* its action on Bax and cytochrome c. HSP60 and HSP70 was demonstrated to have neuroprotective function in arsenic-induced neurotoxicity in red jungle fowl. HSP30, HSP60, HSP70, and HSP90 were elevated in an Avermectin toxicity model in the cardiac tissues of pigeons.

Another member of the HSP family, HSP27, belonging to the small molecular weight HSP family, mediates canonical roles in response to various stresses (Vidyasagar et al., 2012). Initially, HSP27 was characterized as a potent marker for thermal stress, facilitating the effective re-orientation of misfolded proteins (Rogalla et al., 1999; Lelj-Garolla and Mauk, 2006). HSP27 responds to heat shock stimulus in muscle tissues, regulating actin-mediated cytoskeletal dynamics, especially as a capping agent that aids actin polymerization. Furthermore, HSP27-based studies reported that this protein could be a marker not only for thermal stress but also for oxidative and chemical stresses (Vidyasagar et al., 2012). Oxidative stress mediated through the actions of ROS can be resisted effectively by HSP27, which plays a role as a potent anti-oxidant by elevating intracellular glutathione concentrations and limiting the intracellular iron concentration (Arrigo et al., 2005). HSP27 also interacts with apoptosis pathways, thereby mediating an anti-apoptotic effect. It influences the extrinsic pathway of apoptosis by preventing the interaction of activation of apoptosis signal-regulating kinase 1 (Ask 1) with death domain-associated protein (DAXX) (Charette and Landry, 2000). In addition, HSP27 inhibits the intrinsic and mitochondrial apoptotic pathways *via* its action on Bax (apoptotic regulator protein) and cytochrome c (Bruey et al., 2000; Havasi et al., 2008). HSP27 also exerts an anti-apoptotic effect by inhibiting the caspase pathway (Calderwood et al., 2006). These mechanisms can be effective in preventing chemical stress in vital tissues, especially in response to high doses of new generation chemotherapies (Nakashima et al., 2011). Figure 2 depicts the role of HSPs as stress biomarkers.

Elevated levels of HSP60 and HSP70 demonstrated a neuroprotective function in arsenic-induced neurotoxicity in red jungle fowl, *Gallus gallus* (Zhao et al., 2017). The mRNA transcriptional and protein levels of HSP30, HSP60, HSP70, and HSP90 were elevated in an Avermectin (anthelmintic drug) toxicity model in the cardiac tissues of pigeons (Liu C. et al., 2017). Furthermore, elevated levels of HSP90 and HSP70, and glucose-related protein 78 (GRP78), in medullary thyroid carcinoma revealed their potential role in medullary thyroid carcinoma tumor biology, suggesting that they could be developed as biomarkers in the future (Soudry et al., 2017).

Besides heat stress (Baena et al., 2018; Sales et al., 2018), heat shock proteins (HSP) are being evaluated in apoptosis, oxidative stress, inflammatory diseases, cancer (Ikwegbue et al., 2017), virus infection (Shan et al., 2018), bacterial infection (Kim et al., 2018), as immunomodulators (Edkins et al., 2017; Zininga et al., 2018), therapeutics (Skórzynska-Dziduszko et al., 2018), and diagnostics (Lechner et al., 2018; Tang T. et al., 2018). HSPs have diagnostic role in ischemic injury of cardiomyocytes (Santos et al., 2018), inflammatory process of multiple sclerosis (Lechner et al., 2018), early diagnosis of lung cancer (Tang T. et al., 2018), proteome stress (Liu and Zhang, 2018), bipolar disorder (Cheng et al., 2018), and anti-apoptotic agent (Jang et al., 2018).

## Acute Phase Proteins

The innate immune system elicits certain key and prompt responses to defend the body against infection, inflammation, stress, tumor progression, or tissue injury. These systemically activated complex early defensive responses, termed acute phase responses, are promoted by acute phase proteins (APPs), which are serum components, produced primarily by hepatocytes (Jain et al., 2011). Acute phase response (APR) organs include the brain (involved in the increased synthesis of corticotropin-releasing hormone (CRH) and adrenocorticotropic hormone (ACTH), liver (involved in the increased synthesis of metallothionein and antioxidants to restore homeostasis of plasma proteins), bone marrow (increased thrombocytosis and reduced erythropoiesis), adrenal gland (increased cortisol production), and muscle (proteolysis) and adipose cells (altered lipid metabolism) (Robinson et al., 2016). The APR is regulated *via* interleukin 1 receptor antagonist (IL-1RA), IL-10, suppressor of cytokine signaling (SOCS) proteins, and transient expression of APP and their mRNA half-lives (McCormick et al., 2016; Chakrabarti et al., 2018). There are ~200 different APPs in animals. Classification of APPs and their role as diagnostic tool have been reviewed (Jain et al., 2011). APPs are normally secreted under the influence of innate immunity or stress and whose concentrations change upon secretory stimuli. The serum concentrations of positive APPs, such as C-reactive proteins (CRP) and serum amyloid A (SAA), are elevated significantly in response to infections and inflammations (Mittelman et al., 2018). While the serum concentrations of negative APPs, such as albumin and transferrin, decrease in response to infection, inflammation, and stress (Cray et al., 2009). The protein with the highest concentration in serum is albumin, and its reduction or selective loss in biological fluids indicate degenerative conditions of the renal/gastrointestinal system or hepatic insufficiency (Paltrinieri, 2008).

In the 1930s, CRP was the first APP to be described scientifically. This was followed by the discovery of other APPs, including SAA, serum amyloid P (SAP), haptoglobin, ceruloplasmin, fibrinogen, major acute phase protein (MAP), lipopolysaccharide-binding protein (LBP), and α1-acid glycoprotein (AGP) (Kushner, 1982; Petersen et al., 2004; Shamay et al., 2005; Cheung et al., 2008; Ceciliani et al., 2012). Various researchers revealed the influence of these proteins on human health and animal physiology, resulting in them being termed “molecular thermometers” (Murata et al., 2004; Ceron et al., 2005; Cray et al., 2009; Jain et al., 2011). Human CRP is regarded as the primary detector for autoimmune/traumatic/neoplastic conditions (Eckersall et al., 2007). Recent studies in animals indicated the sensitivity of different APPs, suggesting them as strong markers of herd health in large animals (Ganheim et al., 2007; Ceciliani et al., 2012).

**Figure 3:**
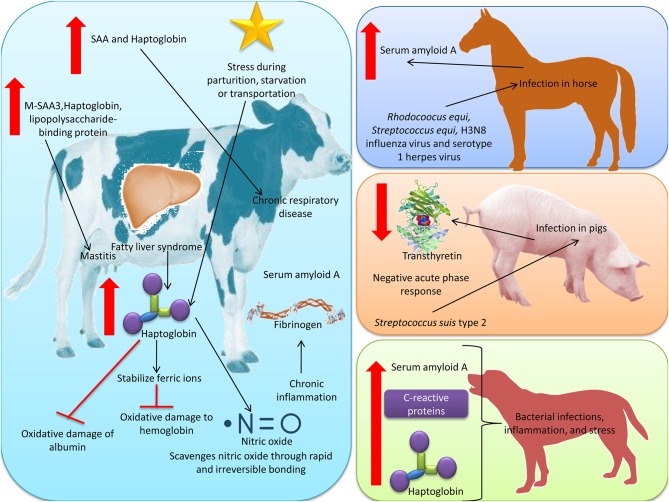
Acute phase proteins as stress biomarkers. Increased level of haptoglobin was noticed in cattle in conditions like fatty liver syndrome and stress induced by parturition, starvation, or transportation. There was also increase in SAA and fibrinogen in cattle during chronic inflammation and associated stress. Haptoglobin exerts its anti-oxidant role by stabilizing ferric irons to prevent oxidative damage to hemoglobin. Haptoglobin protects albumin from oxidative damage by inhibiting the exchange of heme between hemoglobin and albumin. Haptoglobin scavenges nitric oxide through rapid and irreversible bonding, thereby limiting its bioavailability and preventing oxidative damage from reactive nitrogen species. Haptoglobin and SAA are synthesized in the bovine mammary epithelium, and the substantial increase in their secretion into milk was observed during mastitis. SAA has been found to increase in sera of foals during infections with bacteria, i.e., *Rhodocoocus equi* and *Streptococcus equi* and viruses, i.e., H3N8 influenza virus and serotype 1 herpes virus. *Streptococcus suis* type 2 infection in pigs, transthyretin showed a negative acute phase response. CRP is the primary APP in canines, along with haptoglobin and SAA, whose concentrations are elevated in bacterial infections, inflammation, and stress.

APPs have been studied immensely in pets and companion animals. In these species, they have been suggested as the optimum markers for the prognosis of stress conditions and various diseases. Advances in canine and feline medicines have identified several APPs as biomarkers in the prognosis of inflammatory, degenerative, or septic conditions, such as pancreatitis, septic acute kidney injury, mammary tumor, autoimmune disorders, reproductive complications, pyometra, and cardiovascular changes (Hori, 2006; Bayraml and Ulutas, 2008; Gebhardt et al., 2009; Hollinger et al., 2018). Similar to humans, CRP is the primary APP in canines, along with haptoglobin and SAA, whose concentrations are elevated in bacterial infections, inflammation, and stress (Martinez-Subiela et al., 2002; Ceron et al., 2005; McGrotty et al., 2005). In feline species, the serum concentration of α1-acid glycoprotein and SAA are valuable markers of stress, infection, and other inflammations (Verbrugghe et al., 2014). Figure 3 depicts the role of APPs (SAA) as stress biomarkers.

APPs have been proposed as useful indicators for social stresses, such as transportation, mixing, and abrupt weaning, which elicit an acute phase response, especially in young ruminants (Gupta et al., 2007; Herskin et al., 2007). The serum concentrations of ruminant APPs show considerable differences from other animal species, with haptoglobin being the primary ruminant APP. Its already high serum concentration of 20 mg/L can be increased up to 2 g/L within 2–3 days in response to infection, inflammation, and stress (Ceciliani et al., 2012). Increases in haptoglobin levels have been observed in conditions like fatty liver syndrome and stress induced by parturition, starvation, or transportation (Murata et al., 2004; Petersen et al., 2004). Studies on stress-induced variations in APPs in cattle indicated that the concentrations of other APP, such as SAA and fibrinogen, also increase in cattle, especially when exposed to chronic inflammation and associated stress. These APPs were also proposed as indicators of transportation and commingling induced stress in calves (Conner et al., 1998; Arthington et al., 2003). Later, Lomborg et al. (2008) reinforced the findings that APPs, especially SAA and haptoglobin, are potent markers to evaluate transportation or mixing-induced stress in both calves and adult cattle.

Haptoglobin has an anti-oxidant role, especially in ruminant species, preventing them from undergoing oxidative stress, in addition to its standard immune functions including free hemoglobin scavenging and anti-inflammatory actions (Ceciliani et al., 2012). Haptoglobin exerts its anti-oxidant role by stabilizing ferric irons to prevent oxidative damage to hemoglobin. Thus, haptoglobin can protect albumin from oxidative damage by inhibiting the exchange of heme between hemoglobin and albumin (Lim et al., 1998; Buehler et al., 2009). It can also prevent lipid peroxidation damage of vital tissues, particularly in the kidney (Melamed-Frank et al., 2001). Haptoglobin scavenges nitric oxide through rapid and irreversible bonding, thereby limiting its bioavailability and preventing oxidative damage from reactive nitrogen species (Rother et al., 2005).

The well-established influence of APPs upon stimuli resulting from infection, inflammation, and proinflammatory cytokine-mediated responses paved the way for research into their involvement in bovine medicine as markers of various diseases (Eckersall and Bell, 2010). Since certain APPs were detected in milk, their influence has been studied extensively in bovine mastitis, a major bovine inflammation causing considerable economic losses. Haptoglobin and SAA are synthesized in the bovine mammary epithelium, and the substantial increase in their secretion into milk was observed during mastitis (Eckersall et al., 2001). Increases in the milk concentration of the mammary isoform of SAA in ruminants (M-SAA3) have been reported in cows and ewes with mastitis (Winter et al., 2003; Nielsen et al., 2004; Jacobsen et al., 2005; Eckersall and Bell, 2010). In cows with experimentally induced mastitis (using specific microbes, such as *Staphylococcus uberis*), the level of M-SAA3 in milk reached a peak at 6 h, and haptoglobin reached a peak at 10 h after infection (Pedersen et al., 2003; Nielsen et al., 2004). There is an increase in APPs, particularly SAA and haptoglobin in chronic respiratory diseases in calves; in sick calves, concentrations of these parameters were significantly higher in died or euthanized calves compared with calves in improved health status during therapy (Tothova et al., 2010).

SAA has been found to increase in sera of foals during infections with bacteria, i.e., *Rhodocoocus equi* and *Streptococcus equi* and viruses, i.e., H3N8 influenza virus and serotype 1 herpes virus. There was also a statistically significant correlation between SAA serum concentration and severity of clinical signs of the respiratory disease as well as the rectal temperature of the infected animals (Jain et al., 2011).

Following *Streptococcus suis* type 2 infection in pigs, transthyretin (a serum protein which is a negative acute phase reactant) showed a negative acute phase response with serum concentrations reaching a significantly lower level at 2 days following infection compared to serum samples of the healthy pigs (Campbell et al., 2005).

Another potent APP, lipopolysaccharide-binding protein (LBP) which is a liver-derived acute phase protein, has also been reported to show increased concentration in milk during bovine mastitis compared with normal conditions (Zeng et al., 2009). Bovine APPs, such as SAA and haptoglobin, are elevated in the serum during other extra-mammary inflammations, e.g., metritis and interdigital dermatitis. During these extra-mammary inflammation or degenerative changes, no significant alterations in the concentration of these APPs in milk were observed, indicating their specificity in assessing mammary inflammation (Ceciliani et al., 2012). These observations indicated strongly that milk APPs are potent and reliable biomarkers in bovine medicine for the most endemic condition, mastitis, particularly attention subclinical mastitis, which can barely be detected from clinical evidence.

In addition, the effect of transport stress on APPs gene expression in turkey (*Meleagris gallopavo*) revealed upregulation of α1-acid glycoprotein (AGP) and CRP and downregulation SAA and PIT54 in the liver (Marques et al., 2016).

Modern studies are exploring the roles of APPs in both health and productivity, and simultaneously being correlating to stress (Joshi et al., 2018; Miglio et al., 2018). Their diverse roles have enabled them to be potential candidates in theronostics, disease pathogenesis and progression, and prevention. They have been associated with the diagnosis of both infectious and non-infectious diseases including viral diseases (Reczynska et al., 2018), bacterial diseases (tuberculosis) (Santos et al., 2019), cardiovascular diseases (Asleh et al., 2018), acute pulmonary embolism (Zhang Y. X. et al., 2018), pulmonary arterial hypertension (Nakamura et al., 2018), respiratory diseases (Joshi et al., 2018), metabolic disorders in obese children and adolescents (Cura-Esquivel et al., 2018), vascular disorders (Wang S. et al., 2018), chronic inflammatory and neurodegenerative diseases (Luan and Yao, 2018).

These findings revealed the ubiquitous nature of APPs, such that their applications should not be restricted to inflammatory and infectious disease progression and diagnosis. They could be used in a wider range of applications, including monitoring and prognosis of treatment efficacy in humans and animals, and evaluation of the health status of production animals for optimum output; thereby ensuring overall well-being from a public health perspective. However, properly standardized and systematically validated APP assays, such as the Acute Phase Index, are warranted in both the human and animal clinical sectors.

## The Role of Interleukin-22 in the Expression of APR Proteins

Interleukin-22 (IL-22) is implicated in the expressions of acute phase proteins (Liang et al., 2010). IL-22 induced acute-phase protein expression from a HepG2 hepatocellular carcinoma-derived cell line and the liver, with a subsequent increase in the circulating SAA levels (Wolk et al., 2004). IL-22 induced rapid hepatic expression of chemokine ligand, i.e., CXCL1, associated with transient mobilization of neutrophils for systemic ramifications. IL-22 is produced by a subset of T cells, including TH17 and NKT cells. The IL-22 receptor is a heterodimer composed of IL-22R1 and IL-10R2. The expression of IL-22R1 is limited to epithelial and pancreatic acinar cells (Xue et al., 2012). By contrast, IL-10R2 expression is ubiquitous (Sabat et al., 2014; Dudakov et al., 2015). In a recent finding in a mouse model of colitis, an increased expression of serum amyloid A3 has been shown to play a protective role against acute injury through TLR2-dependent induction of neutrophil IL-22 expression (Zhang G. et al., 2018). Expression of anti-microbial peptides for anti-microbial action has also been credited to IL-22 (Narazaki and Kishimoto, 2018).

**Figure 4:**
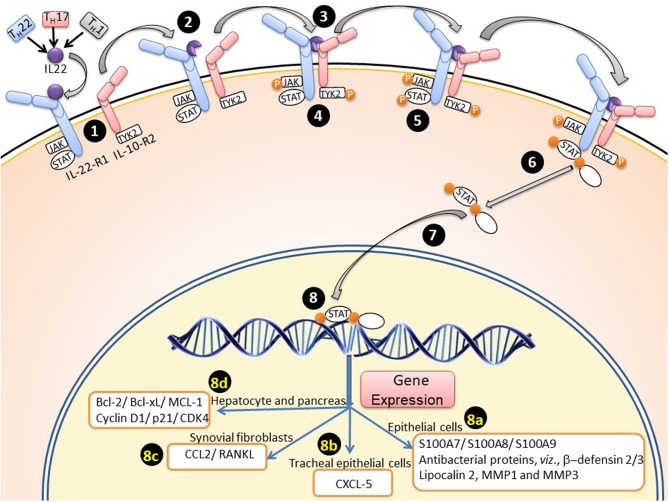
IL-22 signaling pathway for expression of acute phase response proteins. 1. IL-22 receptor is a heterodimer of IL-22R1 and IL-10R2. Initially IL-22 binds to IL-22R1. 2. Upon binding, conformational change in IL-22 enhances its affinity toward IL-10R2. 3. leading to formation of heterodimeric receptor intracellularly, IL-22R1 is associated with JAK1 and STAT3 (STAT3 is recruited through tyrosine-independent recruitment) and IL-10R2 is associated with tyrosine kinase 2 (TYK2). 4. Binding of ligand with receptor initiates the phosphorylation of JAK1 and TYK2. 5. Receptor bound STAT3 is phosphorylated by JAK1. 6. Phosphorylation of STAT3 leads to dimerization of STAT3. 7. Dimerized STAT3 translocates to nucleus; in nucleus, it binds to response elements and regulate associated proteins. 8. STAT3 binds to different genes coding for acute phase proteins by different cells, *viz*. to *viz*. a). Epithelial cell (β-defensin 2, β-defensin 3, lipocalin 2, MMP1 and MMP3, S100A7, S100A8, S100A9 b). tracheal epithelial cells (CXCL5); c). synovial fibroblast (CCL2/RANKL); d). hepatic and pancreatic cells (Bcl-2, Bcl-xL, MCL-1, Cyclin D1, p21, CDK4).

The detailed mechanism of the role of IL-22 in the expression of acute phase response proteins is shown in Figure 4. IL-22 acts on the cells of various organs, including pancreatic cells, liver cells, intestinal epithelial cells, epidermal keratinocytes, respiratory epithelial cells, and synovial fibroblasts (Sabat et al., 2014). In epithelial cells, IL-22 induces the production of anti-bacterial proteins, including β-defensin 2 (BD2), BD3, S100A7, S100A8, S100A9, lipocalin 2, matrix metalloproteinase 1 (MMP1), and MMP3. In hepatocytes and pancreatic cells, IL-22 increases the expression of B-cell lymphoma 2 (Bcl-2), Bcl-xL, myeloid cell leukemia sequence 1 (MCL-1), cyclin D1, p21 (a negative regulator of cell cycle), and CDK4. Specifically, in hepatocytes, it promotes the expression of haptoglobin, LBP, and SAA. In synovial fibroblasts, IL-22 induces CC-chemokine ligand 2 (CCL2) and receptor activator of NF-κB ligand (RANKL) (Sabat et al., 2014). IL-22 expression can act as a sensitive biomarker for identification of allergic children vulnerable to polychlorinated biphenyls (Tsuji et al., 2012).

So, these hormonal, enzymatic or protein biomarkers serve as indicators of inflammation, immunity, stress or related diseases. An overview of these biotic stress markers along with their detection methods is presented in Table 1.

**Table 1:** Biotic stress markers, the associated ailments and their detection methods.

**S. No**.	**Name of molecule**	**Molecule prelude**	**Functions of the molecule**	**Marker to ascertaining**	**Type of stress**	**Diagnosis method**	**Reference(s)**
1.	Malondialdehyde (MDA)	Present in plasma samples; positively correlated to stress	Highly cytotoxic, mutagenic, inhibitors of various enzymes, inhibitor of DNA replication	Ischemic conditions, ocular pathologies, hypertension, Cancer, Alzheimer's disease, respiratory disease	Oxidative stress	Thiobarbitoric acid (TBA) assay	Sordillo and Aitken, 2009; Singh et al., 2013; Wispriyono et al., 2016; Peña-Bautista et al., 2019
2.	Protein carbonyl groups	Chemically stable moieties generated through oxidative cleavage of proteins	Lipid oxidation of cell membrane results in formation of reactive aldehydes and ketone called protein carbonyl groups	Increased levels observed in Alzheimer's disease (AD), rheumatoid arthritis, diabetes, sepsis, chronic renal failure, and respiratory distress syndrome	Oxidative stress	Spectrophotometric assay, ELISA, and one-dimensional or two-dimensional electrophoresis followed by Western blot assay	Dalle-Donne et al., 2003
3.	Isoprostanes	Prostaglandin-like compounds present in plasma, urine or vital tissue samples, formed *via* the free radical-mediated oxidation of arachidonic acid	Reliable *in-vivo* marker of lipid peroxidation	Coronary heart disease, obesity, cancer, genetic disorders, chronic obstructive pulmonary disease (COPD), and acute coronary syndrome	Oxidative stress	Gas chromatography–mass spectrometry, RIA, ELISA	Musiek et al., 2005; Smith et al., 2011; Ferroni et al., 2017; Su et al., 2019
4.	Blood urates	Final product of metabolic breakdown of purine nucleotides	A correlation between blood uric acid level and risk for complications	Gout, kidney stones, cardiovascular disease (CVD), and type 2 diabetes, Parkinson's disease	Oxidative stress (diagnostic and prognostic stress)	Near-infrared (NIR) spectroscopy (use of infrared wavelengths between 1,400 and 1,700 nm)	Cipriani et al., 2010; Stamp et al., 2011; Kim, 2015
5.	3-Nitrotyrosine peptides	Reactive nitrogen intermediates attack on protein bound tyrosine to form 3-nitrotyrosine peptides; a biomarker of nitrogen free-radical species	Expose tissue to reactive nitrogen species	Alzheimer's, Parkinson's, multiple sclerosis, stroke, and cardiovascular (atherosclerosis, myocardial infarction, coronary artery disease, hypertension, and diabetic vasculopathy) diseases	Nitroxidative stress	HPLC Separation and Electrochemical (EC) Detection	Malinski, 2007; Nuriel et al., 2008; Radi, 2013
6.	Liver-type fatty acid-binding protein (L-FABP)	Express in the proximal tubules of the human kidney participating in fatty acid metabolism	Reduce cellular oxidative stress through binding to fatty acid oxidation products, and limiting the toxic effects of oxidative intermediates	Acute kidney injury	Oxidative stress	Sandwich ELISA kit	Ferguson et al., 2010; Kamijo-Ikemori and Kimura, 2015
7.	Endothelin-2	A potent vasoconstrictor	Mainly secreted from endothelial cells	Essential hypertension, hepatorenal dysfunction	Oxidative stress	EIA kit/PCR	Dhawan et al., 2014; Qureshi et al., 2016
8.	Advanced glycation end-products (AGE)	Nucleic acids, proteins or lipids glycated as a result of exposure to reducing sugars	Downstream mediators of tissue injury	Diabetic retinopathy, nephropathy, cataract, neuropathy, cardiomyopathy	Oxidative stress	Fluorescence high-performance liquid chromatography	Calabrese et al., 2007; Singh V. P. et al., 2014
9.	Glutathione peroxidase	Antioxidant selenoprotein enzyme, directly concerned with reduction of free radicals, reducing the oxidative attack	catalyzes the oxidation of glutathione (GSH)	Cancer and cardiovascular disease, systemic lupus erythematosus and anti-phospholipid syndrome	Functional indicator of selenium status	Colorimetric assay	Gheita and Kenawy, 2014; Barry-Heffernan et al., 2019
10.	Superoxide dismutase	Metalloenzyme against reactive oxygen species (ROS) regulating the bioactivity of nitric oxide	Concerned with the direct reduction of ROS into metabolic water and oxygen molecules, thereby preventing the oxidative damages of tissues	Cardiovascular disease, atherosclerosis, ischemia–reperfusion injury	Oxidative stress	ELISA kits using WST method	Fukai et al., 2002; Chen et al., 2018
11.	Heme oxygenase-1 (HO-1)	An increase in HO-1 protein occurs during pro-inflammatory conditions	Up-regulated during selenium (Se) deficiency and suggestive of its role as anti-oxidant compensating for the loss of Se-dependent antioxidants	Ischemia–reperfusion, graft rejection and atherosclerosis	Oxidative stress	ELISA	Trigona et al., 2006; Rücker and Amslinger, 2015
12.	Catalase	Anti-oxidant enzyme	Decompose hydrogen peroxide to water and oxygen	Diabetes mellitus, hypertension, and vitiligo, acatalasemia	Oxidative stress	Formation of a stable and colored carbonato-cobaltate (III) complex	Góth et al., 2004; Hadwan, 2018
13.	Myeloperoxidase	Heme-containing peroxidase expressed mainly in neutrophils	In the presence of H_2_O_2_ and halides, formation of reactive oxygen intermediates, including hypochlorous acid	Diabetes/diabetic retinopathy, obesity, atherosclerosis, cardiovascular diseases	Inflammation	ELISA	Nicholls and Hazen, 2005; Khan et al., 2018
14.	Heat shock proteins (HSPs)	Molecular chaperonins	Stress induced protein denaturation is corrected by refolding and remodeling	Infectious diseases and autoimmune diseases	Indicator of short-term stress like thermal stresses, osmotic stresses, toxicity due to heavy metals, ecological stress due to pollutants	ELISA	Hecker and McGarvey, 2011; Khandia et al., 2017; Tang T. et al., 2018; Silva et al., 2019
15.	Cortisol	Secretion is regulated by the hypothalamo-pituitary-adrenocortical (HPA) system	Stressed conditions increase salivary and blood cortisol	Cardiovascular, metabolic, immunologic, and homeostatic functions	Generalized stress	Competitive lateral flow immunoassay	Ivković et al., 2015; Apilux et al., 2018
16.	Acute phase proteins (APPs)	Innate immune proteins, secreted by hepatocytes	APPS are increased or decreased in response to inflammation	Prostate cancer, bronchopneumonia, chronic inflammation	Apart diseases, environmental stress	RIA and ELISA	Jain et al., 2011
17.	Copeptin	Polypeptide derived from the hypothalamo-pituitary axis-system, as a pre-pro-hormone along with vasopressin and neurophysin II. Employed for assessing the individual stress level in comparison to cortisol. Prognostic accuracy of copeptin in acute illness like sepsis, pneumonia, lower respiratory tract infections and stroke has been analyzed successfully	39-amino acid glycopeptide	Acute myocardial infarction, heart failure, acute exacerbation of chronic obstructive pulmonary disease, lower respiratory tract infections, acute dyspnea, sepsis, hemorrhagic and septic shock, diabetes mellitus, metabolic syndrome, hyponatremia, vasodilatory shock, diabetes insipidus, autosomal dominant polycystic kidney disease (ADPKD), intracerebral hemorrhage, ischemic stroke and traumatic brain injury	Even mild to moderate stress situations alter copeptin level	Sandwich immunoassay	Morgenthaler et al., 2008; Katan and Christ-Crain, 2010; Dobsa and Edozien, 2013

However, their evaluation and interpretation is cumbersome and varies in various conditions or diseases. Sampling, determination, or expression process is relatively tedious. Further, there probable specificity to stress or disease process less likely determines reliability to be used for constant accuracy.

Since biomarkers of stress or related diseases usually vary from condition to condition or organ to organ, efforts are being made to identify a specific and sensitive biomarker that can remarkably diagnose a particular condition or may be characteristic to a group of conditions (Singh et al., 2018). Besides, ease of investigation, safe sampling, and accurate determination can add to the reliability of the biomarker. Though yet in infancy and early stages of investigation, still few specific samples or organ oriented biomarkers are being explored for uniformity and accuracy including salivary, renal, sweat, tears, breath or feces biomarkers. Thus, a detailed account of these salivary or renal biomarkers will be helpful in understanding importance and extent of the diagnostic circumference (Chou et al., 2015; Drozdz et al., 2016; Mortha et al., 2018; Silva et al., 2019).

## Stress Biomarkers in Saliva

Saliva provides an optimum and non-invasive biological source for the quantitative and qualitative assessment of chemical and physiological mediators associated with various conditions, such as stresses, diseases, and injury (Rai and Kaur, 2011). The role of saliva as a mediator can be elucidated from its components, such as digestive enzymes (α-amylase), secretory immunoglobulins for mucosal defense (secretory IgA), innate immune components, such as anti-microbial peptides (lysozyme), and signaling molecules (such as steroidal and peptide hormones) (Wong, 2006; Turner and Ship, 2008). Recent technical advances in the processing and evaluation of salivary components have produced reliable results that increase the possibility of exploiting this biological source, which is comparatively safer, cheaper, and less invasive than its traditional counterparts, such as blood, urine, and peritoneal fluid (Groschl, 2008). Studies suggest that salivary components can be analyzed accurately using specific and sensitive immunological and biochemical techniques, such as RIA, ELISA, and chromatography (Ivković et al., 2015). Reports suggest an intense correlation between exposure to stress and the level of various salivary components, including cortisol, secretory immunoglobulins, enzymes like α-amylase, and chromogranin A. All of the above-mentioned salivary components can be considered as salivary biomarkers of stress in animals (Takai et al., 2004; Takatsuji et al., 2008; Clow et al., 2010). Being a widely accepted salivary biomarker of stress, rapid quantification of cortisol in saliva was investigated as a non-invasive measure to check modulation in stress responses. Apilux et al. (2018) reported the development of a lateral flow immunoassay facility using cortisol-BSA conjugate containing gold nanoparticle labeling in a silver enhancement system to detect cortisol associated with stress from saliva. In addition, oxidative stress markers, including ROS and their mediators, are reported to have been evaluated in salivary samples (Su et al., 2009; Gümüş et al., 2015). Chromogranin A and haptoglobin in saliva are potent candidates for assessing the stress of pigs caused by restraining (Huang et al., 2017).

So far salivary biomarkers have been found to play profound role in diagnosis of many diseases or stressful conditions including cancers (Arantes et al., 2018; Kaur et al., 2018; Khurshid et al., 2018), liver diseases (Abe et al., 2018; Morán and Cubero, 2018), kidney diseases (Maciejczyk et al., 2018b), neurological (Farah et al., 2018) and cardiovascular diseases (Gohel et al., 2018), psoriasis (Asa'ad et al., 2018), systemic lupus erythematosus (Stanescu et al., 2018), and rheumatoid arthritis (Äyräväinen et al., 2018). In one of the very recent review, it has been enumerated that majority of the biomarkers in saliva have diagnostic potential for neoplasms, followed by detection of metabolic disorders and least for systemic disorders (Mortha et al., 2018). A recent study in humans during enduring hot temperature indicated significantly higher levels of biomarkers, such as α-amylase, cortisol, and total proteins in saliva and suggested intensified stressful responses upon exercise during such conditions (Silva et al., 2019). The most commonly used method for estimating these biomarkers is enzyme-linked immunosorbent assay, the most common analytes are immunoglobulins; however, in the recent past, proteomic approaches have enabled discovery of novel salivary biomarkers (Mortha et al., 2018).

**Figure 5:**
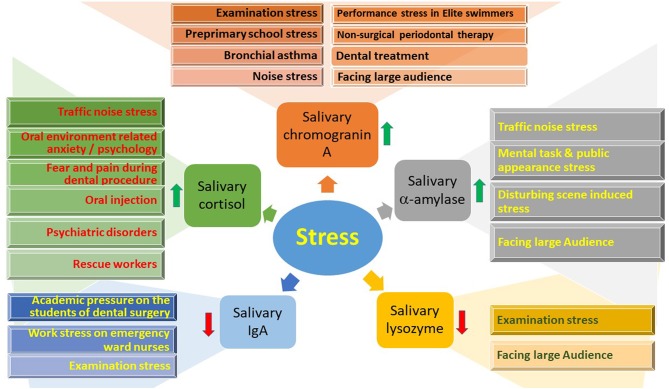
Various stresses affecting the salivary stress biomarkers: cortisol, chromogragranin A and α-amylase are increased whilst salivary IgA and lysozyme are decreased with an increase in level of stress.

Table 2 summarizes the prominent salivary biomarkers associated with stress along with salient research findings indicating their diagnostic potential. Figure 5 depicts various stresses affecting the salivary stress biomarkers.

**Table 2:** Salivary biomarkers and assessment of stress.

**S. No**.	**Salivary marker used to assess the stress**	**Subject(s)**	**Age group**	**Design to evaluate the stress**	**Statistics used/outcome of study**	**Reference**
1.	Salivary chromogranin A	20 males	21–24 years	White noise at 90 dB for 15 min with 15-min-rest periods before and after noise exposure	Friedman's test (*p* = 0.001)	Miyakawa et al., 2006
		5 children	3–5 years	Dental treatment followed by a questionnaire	Two sided Wilcoxon signed rank test (*p* < 0.05)	Mitsuhata et al., 2012
		Professional elite 9 swimmers (6 men and 3 women)	22 ± 2 and 22 ± 4 years, for men and women, respectively	Before and after competition level of stress marker in success and failure athlete	Mann-Whitney comparisons (*p* < 0.05)	Chennaoui et al., 2016
		40 patients	25–60 years	Test of patients with aggressive periodontitis before and after non-surgical periodontal therapy	*post hoc* test (*p* < 0.001)	Lihala et al., 2019
		62 (34 males, 28 females)	57.6 ± 13.4	To fill the SF-36 questionnaire indicative of quality of life (QOL)including the parameters of Peak Expiratory Flow, role physical and role emotional in patients with Bronchial Asthma	Kruskal-Wallis rank test (*p* < 0.05)	Hoshino et al., 2008
		56 preprimary school students	2.5–5 years	Stress marker correlation with self-structured preprimary stress questionnaire (PPSQ)	One-way ANOVA (*p* < 0.001)	Jena and Mohanty, 2016
2.	Salivary IgA and lysozyme	132 female emergency department and general ward nurses	Not given	Stress marker correlation with mental health professionals stress scale (PSS)	Test not given (*p* = 0.001)	Yang et al., 2002
3.	Salivary IgA	514 nurses	22–43 years	33 data points collected for immunological measures to find out association with stress and job in a voluntary job stress survey	ANOVA (*p* < 0.01)	Lee et al., 2010
		130 dental students	22.4 ± 2.5	A 38-item dental environmental stress (DES) questionnaire and a 4-point perceived stress scale to identify self-perceived stress levels (stress due to academic pressure)	A two sample independent *t*-test, Scheffe's test, partial correlation coefficient (Pearson's *r* = −0.30, *p* = 0.0001)	Ng et al., 2004
4.	Salivary amylase and cortisol	20 participants	20–44 years	Exposure to naturalistic traffic noise samples containing 75 dB (LA, eq) for 20 min *via* a loudspeaker system	Test not given (*p* < 0.01)	Wagner et al., 2010
5.	α-amylase	30 healthy young men	19–28 years	Participants subjected to Trier Social Stress Test (TSST) consisting of a mental arithmetic task and free speech in front of an audience	Analyses of variance (ANOVA) (*p* < 0.001)	Nater et al., 2006
		83 healthy volunteers	20–27 years	Stress induced by showing scenes of injection into the eyeball and incision of the cornea with scissors for 15 min	Unpaired *t-*test; Pearson's correlation coefficient (*r*); Positive correlation between the amylase level and the State-Trait Anxiety Inventory (personal anxiety test) score (*r* = 0.535; *p* < 0.01)	Takai et al., 2004
		100 subjects (50 chronic stressed + 50 healthy)	Over 18 years	Subjects were asked to fill a psychometric questionnaire	Non-parametric Mann-Whitney U Test (*p* = 0.002)	Vineetha et al., 2014
6.	Cortisol	170 children	11–14 years	Revised Children's Manifest Anxiety Scale (RCMAC) Questionnaire was filled to evaluate child anxiety in connection to oral environment and its connection to the general psychological status of children	T-standard evaluation (X50; SD10) by linear transformation of the data, step algorithm and Single correlations (*p* < 0.05)	Rashkova et al., 2010
		20 children	4–8 years	Evaluation of fear and pain stress during dental procedure (10 min before, during the procedure, and 30 min after excavation of caries with rotary hand piece an tooth extraction)	Paired *t*-test, two independent sample *t*-tests, and analysis of variance (ANOVA) (*p* < 0.05)	Patil et al., 2015
		30 patients with clinically and histopathologically proven cases of oral lichen planus with matched healthy individual	19–69 years	Assessment of intensity of burning sensation was determined using a Visual Analog Scale with psychological evaluation was done with depression, anxiety, and stress scale	Student's *t*-test (*p* < 0.001)	Shah et al., 2009
		1,755 adolescents	13–18 years	The National Comorbidity Survey-Adolescent Supplement questionnaire to measure stress reactivity in adolescents with psychiatric disorders	Quantile regression models; adolescents with mood and/or anxiety disorders have high cortisol levels	Brown, 2016
		65 rescue workers	34–59 years	Workers subjected to Psychiatric self-rating scale General Health Questionnaire (GHQ-28) measuring psychiatric health, Impact of Events Scale (IES) and Post-Traumatic Symptom Scale (PTSS) measuring post-traumatic symptoms	Non-parametric Spearman rank correlation coefficients; correlation between evening salivary cortisol and anxiety (*p* < 0.005), depressive symptoms (*p* < 0.01), post-traumatic symptoms with avoidance behavior (*p* < 0.005)	Aardal-Eriksson et al., 1999
7.	Salivary lysozyme	17 participants in final year examination (7 males + 10 females)	20–26 years	Sample taken before and after the examination and assessed for study	paired student's *t*-test (*p* < 0.05)	Perera et al., 1997
8.	Chromogranin A and salivary lysozyme, Salivary amylase	26 female professors	7.2 ± 3.0 years	State-trait anxiety inventory (STAI) was used to assess personal anxiety while delivering lecture in front of 200 students	Two-way repeated measures ANOVA; Salivary lysozyme (*p* < 0.05); Chromogranin A (*p* < 0.05); Salivary amylase (*p* < 0.05)	Filaire et al., 2010
9.	Chromogranin A and IgA	15 female students appearing in nursing exam	21–26 years	Sample taken 10 min before exam, and immediately after the exam and 2 h post-exam	Student's *t*-test (*p* < 0.05)	

## Renal Stress Biomarkers

Blood urea nitrogen (BUN) and creatinine are the most commonly used biomarkers to assess the functional status of the kidney and can be measured easily and inexpensively in serum. Urea and creatinine are the normal metabolic end products of dietary and tissue-derived proteins in healthy humans and animals. They are cleared from the circulation *via* the kidneys. Even though they are potent markers for assessing renal insufficiency in mammals, a range of serum levels reflecting different pathophysiological statuses resulting from many non-renal etiologies have been observed (Tesch, 2010). Urea and creatinine levels in the serum are influenced by non-renal causes, such as dietary intake of protein, dehydration due to temperature and humidity stresses, defective liver function, bleeding in the gastrointestinal tract, and chronic steroid use. BUN is customarily quantified in serum by an enzyme/oxidation-reduction assay; however, BUN assays often misjudge renal function because of interfering chromogens (Waikar and Bonventre, 2006). Different assays are employed to quantify creatinine levels in serum and urine, such as the creatininase method and HPLC; however, the Jaffe rate reaction is used most commonly because it is cheap and easy to perform (Schwartz et al., 2009).

Renal oxidative stress responses can also be measured using serum and urine biomarkers in human patients and animal models. One such renal oxidative stress marker is 8-OH-dG, which is a metabolically stable product of the oxidation of guanine from nucleic acids. The 8-OH-dG level increases in urine following renal oxidative stress, which can be detected by standard enzyme immunoassays (Dounousi et al., 2006). Cystatin-C, a cysteine protease inhibitor, is another reliable biomarker to assess renal function. Cystatin-C is released regularly into the circulation from nucleated cells and is reabsorbed normally by kidney tubules, followed by its catabolism (Curhan, 2005). Thus, the increased concentration of cystatin-C in blood and serum samples is highly sensitive to assess renal impairment and compared with creatinine, it is a very potent indicator of acute renal injuries because of its shorter half-life (Herget-Rosenthal et al., 2004). Although its quantification can be affected by steroid therapy and thyroid dysfunctions, immunonephelometry and enzyme immunoassays can be used effectively to measure the serum cystatin-C concentration.

Increased lipid peroxidation during oxidative stress leads to the production of several chemo-mediators, in which 8(F2a)-isoprostane and 4-hydroxy-2-nonenal can be detected not only in serum but also in urine samples by HPLC or immunoassays. Their increased serum concentrations are indicative of chronic renal disorders (Calabrese et al., 2007; Tesch, 2010). Besides these conditions, urinary excretion levels of 8-iso-prostaglandin (PG) F_2α_ has also been described for a strong association with coronary problems and diabetes. Higher urinary 8-iso-PGF_2α_ levels are being correlated to an increase in necrotic plaque formation and coronary culprit lesions in diabetes patients, especially with acute coronary problems (Su et al., 2019). Also, renal oxidative stress can be measured using markers, such as 3-nitrotyrosine peptides, which are stable nitrated peptides produced through the action of peroxynitrites upon protein tyrosine residues (Radi, 2013). The effectiveness of 3-nitrotyrosine peptides, present in urine and serum samples, as potential biomarkers of renal oxidative and nitrosative stress, has been reported in several studies (Nemirovskiy et al., 2009). Their levels can be determined accurately using liquid chromatography and mass spectroscopy (Radabaugh et al., 2008).

Certain modified proteins, such as advanced glycation end products (AGEs) are increased in the circulation as a consequence of oxidative stress, especially in diabetes and uremia. AGEs, together with pentosidine, can also be deposited in kidneys, resulting in renal cellular dysfunction and/or renal damage (Calabrese et al., 2007). Thus, elevated levels of these compounds, which can be assessed by ELISA/HPLC, are effective markers of oxidative stress and help to predict the development of nephropathic conditions in diabetic patients. Liver-type fatty acid-binding protein (L-FABP), released by proximal tubular cells, has been identified as a potent marker of hypoxic stress-induced renal damage. Increased urinary secretion of L-FABP correlates with a declining renal function that might be caused by acute or chronic renal tubular injury (Portilla et al., 2008; Ferguson et al., 2010; Kamijo-Ikemori and Kimura, 2015).

Potent biomarkers for sensitizing renal injury include albumin, N-acetyl-β-D-glucosaminidase, kidney injury molecule-1, and exosomal transcription factors (Coca et al., 2008). The excretion rate of albumin into urine is considered as a routine early biomarker to assess the renal injury, such that an increase in the urinary albumin level (albuminuria) indicates the possibility of renal dysfunction (Meijer et al., 2010). N-acetyl-beta-D-glucosaminidase is a proximal renal tubule-derived lysosomal enzyme that has been identified as a sensitive marker for both acute and chronic renal injury (Han et al., 2008; Jungbauer et al., 2011). Potential sourcing of urinary biomarkers that can be employed for the improved diagnostic tests is identified as pathways involved in kidney damage, oxidative stress and low-grade inflammatory changes associated with atherosclerosis/vascular damage (Matheson et al., 2010). Several studies have revealed that in case of chronic kidney diseases the level of various stress markers/oxidative stress markers, such as products of oxidation of protein (advanced products), isoprostanes, and malondialdehyde increase along with a reduction in the concentration of anti-oxidants. Such markers are valuable to detect atherosclerosis (and the rate of its progress) associated with disturbed kidney functions (Gosmanova and Le, 2011; Sung et al., 2013; Tucker et al., 2013; Drozdz et al., 2016).

Biomarkers of renal dysfunction, such as transferrin, type IV collagen, N-acetyl-beta-D-glucosaminidase, etc., inflammatory markers, such as orosomucoid, tumor necrosis factor-alpha, monocyte chemoattractant protein-1 (MCP-1), vascular endothelial growth factor, as well as oxidative stress markers, such as 8-OH-dG can be superior candidates in the detection of renal disorders including nephropathy and associated cardiovascular disease (Matheson et al., 2010; Chou et al., 2015). Valuable biomarkers indicative of acute kidney injury (AKI) are insulin-like growth factor-binding protein 7 (IGFBP7), tissue inhibitor of metalloproteinases-2 (TIMP-2); their levels in urine need to be monitored on urgent basis (Kimmel et al., 2016). Another proximal renal tubule expressed transmembrane protein is kidney injury molecule-1; its increased concentrations in urine, which can be detected effectively using immuno-enzymatic and immunochromatographic techniques, correlate highly with proximal renal tubular injury (Van Timmeren et al., 2007; Vaidya et al., 2009). Several pathological invasions activate the expression of exosomal transcription factors (ETFs) that are contained in the exosomal vesicles of renal tubular epithelial cells. Assessment of these ETFs in urine by transcriptome sequencing and Western blotting could be effective to diagnose acute renal injuries, as well as in monitoring disease progression (Zhou et al., 2008). Taken together, the accumulated biochemical and immunological data for stress-induced renal dysfunctions and associated biomarkers provide a sound foundation for further study of the molecular mechanisms of disease progression, prognosis, and therapeutic evaluation.

**Figure 6:**
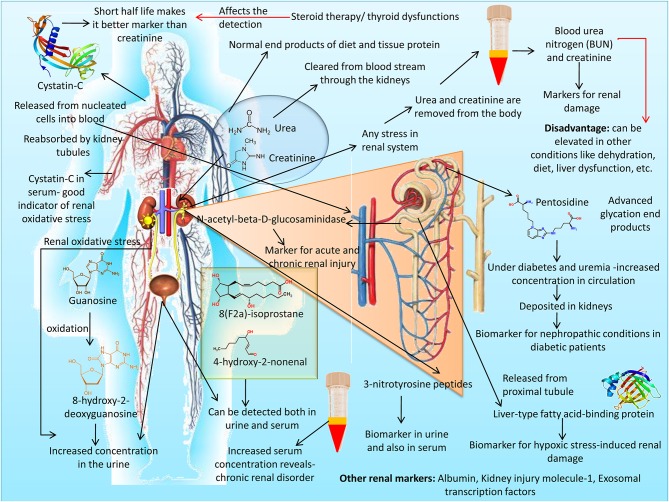
An overview on renal stress biomarkers: various biomarkers, such as urea, creatinine, 8-hydroxy-2-deoxyguanosine, cystatin-C, and others are used to assess the status of renal system.

Certain biomarkers including estimated glomerular filtration rate (eGFR), serum creatinine, blood urea, cystatin-C, and proteinuria or albuminuria can be employed for diagnosis of diabetic nephropathy (DN) (Campion et al., 2017; Moledina, 2019). Urinary heat shock protein 72 (uHSP72) was recently found to be a novel biomarker for detection of DN and was also known to be a biomarker for acute kidney injury (El-Horany et al., 2017). Other novel renal biomarkers are neutrophil gelatinase-associated lipocalin, urinary activin A, kidney injury molecule-1, monocyte chemotactic peptide-1, IL-18, netrin-1, cycle arrest markers, endogenous ouabain, selenium-binding protein 1, and BPIFA2 marker (Arsalan et al., 2018; Beker et al., 2018; Takahashi S. et al., 2018; Yimer et al., 2018). An overview of renal stress biomarkers to assess the status of the renal system is depicted in Figure 6.

Hence, salivary and urinary biomarkers have found promising scope for future applications in biomedical science in diagnosing stress related and pathophysiological diseases and disorders. Being natural secretory or excretory products and not involving any invasive protocols makes diagnostic procedure convenient besides overcoming ethical issues of sampling or investigation procedures. Though numerous biomarkers have already been identified in these natural secretions and many proving beneficial of diagnostic value, still much needs to be investigated for covering broad range problems with accuracy and repeatability so that they can be utilized in stresses, diseases or disorders. Many may prove effective as prognostic and therapeutic markers enabling forecasting or progression of the disease and monitoring the effect of therapy on the management of disease as discussed ahead.

## The Utility of Biomarkers in the Clinical Illness: Prognosis and Therapy Guidance/Selection

Various critical molecular signatures have been identified in relation with stress associated ailments clinical, but for their recommendation as a unique biomarker for each condition depends on the clinical relevance as well as cost-effectiveness, sensitivity, and specificity of diagnostic tools. Table 3 depicts an overview of various biomolecules partaking diagnostic/prognostic values along with their association with various ailments. Scientific advances mainly paved for the exploration of oxidative stress related biomarkers for using clinically, but nowadays more focus are being paid on the use of non-invasive biological samples wherein numerous studies have proposed the use of saliva and urine stress biomarkers in the prognosis and diagnosis of infectious, non-infectious and metabolic diseases (Frustaci et al., 2012; Lindsay and Costello, 2017).

**Table 3:** Overview of various biomolecules having diagnostic/prognostic values along with their association with various ailments.

**S. No**.	**Molecular marker**	**Molecule category**	**Vita of the molecule**	**Association with ailment**	**Marker for diagnosis/prognosis**	**Detection module**	**Reference(s)**
1.	Glutamate	Mitochondrial phosphate-dependent glutaminase converts glutamine to glutamate and ammonia	Essential to protein synthesis, muscle growth, regulation of acid-base balance in the kidney, ureagenesis in the liver, hepatic and renal gluconeogenesis, oxidative fuel for the intestine and cells of the immune system, precursors of neurotransmitter synthesis, of nucleotide and nucleic acid synthesis and of glutathione production	Visceral obesity and associated metabolic alterations	Diagnostic marker for visceral obesity and altered metabolism	Targeted metabolomics using the Absolute IDQ kit p180	Maltais-Payette et al., 2018
2.	Chromogranin-A (CgA)	Present in the secretory granules of most endocrine and many neuroendocrine cells	Considered to serve as precursor molecule for biologically active peptides	Neuroendocrine tumor	Diagnostic and prognostic marker for survival	Solid-phase, two-site immuno-radiometric assay, the CGA-RIA kit	Deftos, 1991; Bílek et al., 2019
3.	Urinary 8-hydroxy-2-deoxyguanosine (8-OH-dG)	An oxidized nucleoside of DNA	Excreted in the urine upon DNA repair	Cancer, atherosclerosis, diabetes, diabetic nephropathy and retinopathy	Diagnostic and prognostic marker	Alkaline comet assay	Wu et al., 2004; Franken et al., 2017
4.	Liver-type fatty acid-binding protein (L-FABP)	Express in the proximal tubules of the human kidney participating in fatty acid metabolism	Reduce cellular oxidative stress through binding to fatty acid oxidation products, and limiting the toxic effects of oxidative intermediates	Acute kidney injury	Diagnostic marker effective in estimating severity of renal injury/oxidative stress biomarker	Sandwich enzyme-linked immunosorbent assay (ELISA) kit	Ferguson et al., 2010; Kamijo-Ikemori and Kimura, 2015
5.	Renal Wilms' tumor-1 (WT-1)	WT-1 is constitutively expressed on podocytes in healthy adult kidneys	Maintain normal podocyte function	Focal segmental glomerulosclerosis, steroid-sensitive nephrotic syndrome, acute kidney injury	Prognostic marker of podocyte injury	Differential centrifugation followed by lysis and immunoblotting	Zhou et al., 2008, 2013
6.	N-acetyl-beta-D-glucosaminidase	Lysosomal enzyme abundantly present proximal kidney tubule cells	Indicative of renal tubular function, patients with tubular and interstitial renal impairment, the total activity of urinary NAG is elevated	Chronic heart failure, acute kidney injury	Prognostic for mortality and rehospitalization for heart failure, renal impairment	Fluorimetric assay based on the fluorescent 4-methylumbelliferyl-N-acetyl-β-D-glucosaminide substrate or spectrophotometric method is based on highly soluble and stable 4-nitrophenyl-N-acetyl-β-D-glucosaminide as substrate	Skálová, 2005; Han et al., 2008; Jungbauer et al., 2011
7.	D-serine	Formed through serine racemase enzyme mediated conversion of L-serine	Anti-depressant properties	Schizophrenia, Alzheimer's disease	Prognostic biomarker for anti-depressant response to ketamine	High performance liquid chromatography/ amperometric, biosensor-based method	Papouin and Haydon, 2018; MacKay et al., 2019
8.	Osteocalcin	Secreted solely by osteoblasts	Pro-osteoblastic, responsible for bone mineralization and calcium ion homeostasis	Chronic rheumatic diseases, bone metastases	Prognostic marker for skeletal metastasis marker	ELISA	Arai et al., 1992; Mishra, 2017; Anderson et al., 2018
9.	Cathepsin-D	Ubiquitously expressed lysosomal aspartic protease	Protein degradation and cell death and regulation of trypsinogen activation	Cystic fibrosis, pancreatitis *via* inflammatory cells	Prognostic marker for poor prognosis in glioma patients	Real-time -reverse transcription-PCR analysis	Fukuda et al., 2005; Mishra, 2017
10.	Urease, ammonia/urea (breath)	Neutralize stomach acid by producing ammonia from urea diffusing from the blood	Hydrolyze urea, to form ammonia and carbon dioxide	Gastritis, peptic ulcer and gastric cancer	Diagnostic marker for the presence of *H. pylori*	Rapid urease test, urea breath test	Mishra, 2017; Graham and Miftahussurur, 2018
11.	Calcitonin gene-related peptide (CGRP), and substance P (SP)	Pronociceptive role	Involved in development of pain and hyperalgesia	Colonic hypersensitivity	Diagnostic Marker of neurogenic inflammation	Capillary Isoelectric Focusing (CIEF) immunoassay	Delafoy et al., 2006; Mishra, 2017; Jasim et al., 2018
12.	Brain-derived neurotropic factor (BDNF)	Neurotrophin having role in neuronal survival and development	Involved in mechanism of hyperalgesia	Upregulated and associated with pain in chronic pancreatitis	Diagnostic marker of chronic pancreatitis	Immunohistochemistry	Zhu et al., 2001
13.	Galectin-3	A member of β-galactoside binding lectins	Activates a variety of profibrotic factors, promotes fibroblast proliferation and transformation, and mediates collagen production	Fibrotic diseases, cardiac disorders, asthma, atherosclerosis, atopic dermatitis	Prognostic biomarker in patients with heart failure	ELISA, BGM Gal-3 assay; RCHITECT Gal-3 assay	Gehlken et al., 2018; Sciacchitano et al., 2018
14.	Mucin	Prolines, threonines and serine rich proteins that contain tandem repeat motifs	A physical barrier limiting damage to the epithelium and attenuates activation of innate and adaptive immune responses	Deficiency causes inflammation of the colon and superficial erosions consistent with ulcerative colitis	Diagnostic and prognostic marker of gastrointestinal disease, cancer	Solid-phase sandwich ELISA	Kufe, 2009; Chen et al., 2015; Celi et al., 2018; Bademler et al., 2019
15.	Neutrophil gelatinase associated lipocalin and cystatin C	Expression is induced in liver, spleen and immune cells in response to ischemic damage or other kidney insult; cysteine protease inhibitor expressed by nucleated cells	–	Indicative of kidney damage	Diagnostic biomarker for tubular damage markers	ELISA	Siddiqui et al., 2017
16.	Lactoferrin	Member of the transferrin family of iron-binding glycoproteins	Component of the innate immune system, binds to iron with high affinity and thus control inflammation	Obesity, type 2 diabetes, and cardiovascular diseases	Non-specific diagnostic marker of inflammation	Immunoassay	Mayeur et al., 2016
17.	Tumor M2-pyruvate kinase (Tumor M2-PK)/M2-pyruvate kinase	Tissue-specific isoenzymes are replaced by M2-PK	In tumor cells is a shift from the tetrameric form to a nearly inactive dimeric form occurs	Increased in some human cancers, diabetes mellitus, coronary heart disease, chronic renal failure	Prognostic biomarker for pancreatic cancer, GIT stress/diseases	ELISA	Siddiqui et al., 2017; Bandara et al., 2018
18.	Catestatin (CST)	Peptide derived from the neuroendocrine protein chromogranin A	Autocrine inhibitor of catecholamine secretion, regulates hypertension	Cardiovascular disorders	Diagnostic marker of psychological stress associated with increased mortality in heart patients	ELISA	Mahata et al., 2018
19.	Fecal secretogranin	Proteins present in secretory cells of the enteric, endocrine, and immune systems	Reflect activity of enteric, endocrine, and immune systems	Ulcerative colitis, irritable bowel syndrome	Prognostic marker	Commercial radioimmunoassay	Ohman et al., 2012; Magnusson et al., 2018
20.	Cystatin-C	Member of group of cysteine protease inhibitors	Produced mainly through nucleated cells	Chronic kidney disease	Diagnostic marker for glomerular filtration	Particle-enhanced turbidmetric immunoassay	Onopiuk et al., 2015; Ogawa-Akiyama et al., 2018
21.	Urinary activin A	Member of the TGF-beta superfamily, is correlated with the degree of tubular damage	stimulated by inflammatory mediators	Ischemic kidneys	Diagnostic biomarker for acute kidney injury	ELISA	Takahashi S. et al., 2018
22.	Cardiac troponin (cTn)	Cardiac-specific proteins that are part of the troponin complex part of contractile apparatus	Released in blood followed by an acute myocardial infarction (AMI) and other types of acute myocardial injury	Cardiac injury	Diagnostic biomarker for acute myocardial infarction	Troponin I assay	Hammarsten et al., 2018
23.	Visfatin	An endocrine, autocrine as well as paracrine peptide	Participate in enhancement of cell proliferation, biosynthesis of nicotinamide mono- and dinucleotide and hypoglycemic effect.	Endometrial cancer, diabetes	Diagnostic marker	Multiplex fluorescent bead-based immunoassays	Adeghate, 2008; Baldassarre et al., 2018; Cymbaluk-Płoska et al., 2018
24.	Interleukin-6	A pleiotropic, pro-inflammatory cytokine	Play an important role in inflammation, immunity, reproduction, metabolism, hematopoiesis, neural development, bone remodeling and angiogenesis	Albuminuria, retinopathy, and cardiovascular disease	Diagnostic and prognostic marker	SignaturePLUS Protein Array Imaging and Analysis System	Baker et al., 2018; Vainer et al., 2018
25.	Fibrinogen	A glycoprotein that enzymatically converted to fibrin and subsequently forms fibrin-based blood clot	Traditional markers of inflammation	Chronic kidney disease	Prognostic marker	Immunonephelometry	Baker et al., 2018
26.	C-reactive protein (CRP)	An acute phase reactant of the pentraxin family	During pathogen-independent inflammation, CRP binds to DNA and histones and scavenges the nuclear materials released from damaged circulating cells to activate innate immune cells	Solid tumor	Prognostic marker	Turbidmetric immunoassay	Baldassarre et al., 2018; Shrotriya et al., 2018
27.	Natriuretic peptide	A polypeptide hormone secreted by heart muscle cells	Reduce blood pressure, diuretic, sympathetic outflow, and vascular smooth muscle and endothelial cell proliferation	Acute (decompensated) and chronic heart failure, renal disease, hyperthyroidism, pulmonary diseases	Diagnostic and prognostic marker	Single-epitope sandwich assay	Tamm et al., 2008; Pandit et al., 2011; Vodovar et al., 2018
28.	Carbohydrate antigen 125 (CA 125)	A glycoprotein, product of MUC16 gene	Produced as a consequence of mechanical stress, such as fluid overload/serosal effusions and/or inflammation	Ovarian cancer, heart failure	Prognostic marker	Quantitative ELISA kit	Polineni et al., 2018; Stanciu et al., 2018
29.	Endothelin-1	A potent vasoconstrictor peptide	Mainly secreted from endothelial cells	Systemic sclerosis, spontaneous cardiac or respiratory diseases	Diagnostic and prognostic marker	Radioimmunoassay (RIA)	Tessier-Vetzel et al., 2006; Odler et al., 2018
30.	Angiogenin	Member of the vertebrate-specific secreted ribonuclease A superfamily	Induce blood vessel formation	Colorectal cancer, acute myeloid leukemia, multiple myeloma, myelodysplastic syndromes cardiovascular diseases	Diagnostic and prognostic marker	ELISA	Yu et al., 2018
31.	β2-Microglobulin	100-amino acid protein encoded by gene present on chromosome 15 in human	Tertiary structure is similar to the constant domain of the immunoglobulins and associate with human leukocyte antigen I (HLA-I) on the surface of all nucleated cells. The interaction is essential to antigen presentation	Acute kidney Injury, familial hypercatabolic hypoproteinemia, solid organ malignancies, lymphoproliferative disorders, such as myeloma and chronic lymphoblastic leukemia, and many autoimmune diseases, tubulointerstitial nephritis and uveitis (TINU) syndrome	Diagnostic marker	BN ProSpec Nephelometer	Hettinga et al., 2015; Lu et al., 2018

Oxidative stress-related biomarkers are important to detect autism in patients with autism spectrum disorders (ASDs). A decline in blood levels of reduced glutathione (27%), glutathione peroxidase (18%), methionine (13%), and cysteine (14%), and increased concentrations of oxidized glutathione (45%) relative to controls were indicative; whereas, superoxide dismutase, homocysteine, and cystathionine showed no association with ASDs (Frustaci et al., 2012). Furthermore, oxidative stress has been postulated as a key factor in the pathogenesis of neurodegenerative disorders, such as amyotrophic lateral sclerosis (ALS), Parkinson's disease (PD) (Medeiros et al., 2016), and Alzheimer's disease (AD) (Mander et al., 2016; Niedzielska et al., 2016). The presence of neurodegenerative diseases is revealed by elevated levels of oxidative stress biomarkers and by decreased levels of anti-oxidant defense biomarkers in the brain and peripheral tissues. Some oxidative stress markers, including, F2-isoprostanes and isofurans in plasma, are related to excess cardiovascular risk and are very common in patients with end-stage renal disease (Rivara et al., 2017). In addition, lipid oxidation could be a promising stress biomarker to diagnose systemic lupus erythematosus (Hu et al., 2016). Assessment of lipid peroxidation markers in non-invasive biological samples, especially saliva, has been validated through novel methods including ultrasound-assisted liquid-liquid semi-microextraction (UA-LLsME), tandem mass spectrometry and ultra performance liquid chromatography. The findings strongly recommend the adoption of such analytical methods in determining the level of lipid peroxidation as well as its potential as a potent oxidative stress marker and correlation with neurodegenerative changes (Peña-Bautista et al., 2019).

Furthermore, levels of the stress biomarker, MDA were significantly selective for cases of extrapulmonary tuberculosis compared with healthy controls (*p* < 0.05) (Goyal et al., 2016). Besides, the therapeutic efficacy of various molecules, such as coenzyme Q10 upon systemic diseases, such as coronary artery disease is also being assessed based on their impact over oxidative stress biomarkers (Jorat et al., 2019). Moreover, salivary MDA has been recognized as an important stress biomarker in systemic and oral diseases (Khoubnasabjafari et al., 2016). In another study, in the first month after birth, elevated F2-isoprostanes, produced through two distinct pathways simultaneously in the disease state predict poor respiratory outcomes and neuro-developmental risk in very preterm infants (Matthews et al., 2016). IL-6 and IL-8 have been reported to be used as potential biomarkers for diagnosis of oral pre-malignant lesion and oral carcinoma (Khyani et al., 2017).

Stress biomarker research reported that oxidative biomarkers could be promising approaches for enantioselective toxicity control of chiral pesticides (Ye et al., 2017). By contrast, the enzymatic anti-oxidant defense system, which is damaged in recurrent aphthous stomatitis patients with active lesions, has been proposed to have an important role in its pathogenesis and could represent the best stress biomarker in this situation (Zhang Z. et al., 2017). Plasma protein-bound di-tyrosines were designed as oxidative stress biomarkers in patients with end-stage renal disease on maintenance hemodialysis (Colombo et al., 2017). Also, a strong association between oxidative stress and anti-oxidant biomarkers in the circulating, cellular, and urinary anatomical compartments in Guatemalan children from the western highlands was reported (Soto-Mendez et al., 2016). The study revealed that excessive oxidation is suggested to be associated with an increase in the urinary biomarkers of oxidative stress F2-Iso and 8-OHdG andurinary excretion of oxidative biomarkers associates directly with the activity of antioxidant enzymes and inversely with the vitamin concentration. Studies on the relationship between lipid peroxidation biomarkers offer a promising research line that need to be developed with the aim to help clinicians in early disease diagnosis, effective treatment initiation and reliable disease monitoring (García-Blanco et al., 2017b).

Oxidative stress enhanced lipid peroxidation (LPO) leads to the accumulation of 4-hydroxy-2-nonenal that ultimately produces exocyclic etheno-DNA adducts, which are strong pro-mutagenic DNA lesions. Biomonitoring of etheno-DNA adducts from tissues, white blood cells and urine using ultra-sensitive detection methods are promising tools for the prognosis of malignancy, the efficacy of chemopreventive and outcome of therapeutic interventions (Bartsch et al., 2011).

Dietary anti-oxidants are inversely related to oxidative stress biomarkers among men with prostate cancer (Vance et al., 2016). Men diagnosed with prostate cancer are reported to have increased oxidative stress and lower antioxidant enzyme activity (Arsova-Sarafinovska et al., 2009). Furthermore, increased levels of urinary oxidative biomarkers, such as 8-hydroxydeoxyguanosine (8-OH-dG), could be considered the best oxidative stress biomarkers in metal oxides nanomaterial-handling workers (Liou et al., 2016). Increased level of oxidative stress markers, such as malondialdehyde and a decreased level of anti-oxidative markers, such as superoxide dismutase and glutathione peroxidase in the serum or the aqueous humor is suggested to be reliable aids in the diagnosis of glaucoma as reported by Benoist d'Azy et al. (2016), through a systematic review and meta-analysis. Therapeutic efficacy of molecules, e.g., ascorbic acid in ameliorating oxidative stress has been evaluated based on the plasma levels of markers including albumin, malondialdehyde, and superoxide dismutase (Yimcharoen et al., 2019).

Biomarkers associated with post-traumatic stress disorder (PTSD) include endocrine as well as molecular biomarkers. Assessment can be done at the genetic level (DNA or single nucleotide polymorphism biomarkers), at the level of expression of a gene, e.g., RNA biomarkers, the protein levels, such as peptide as well as proteins as biomarkers, etc. (Schmidt et al., 2013). Assessment can also be done at the epigenome level to program the genomic activity by various ways, such as methylation of DNA, modifications of histone, and RNA interference, otherwise termed as epigenetic biomarkers. Imaging biomarkers are also associated with PTSD whose assessment can be done by either structural method, such as magnetic resonance imaging (MRI) or functional method, such as MRI/fMRI (Berger et al., 2009; Schmidt et al., 2013; Bisson et al., 2015). Certain common biomarkers are critical in inducing morphological alteration in tissues, for instance, experimentally induced febrile seizures were reported with morphological changes in the brain, such as dentate gyrus which is mainly elicited by corticosterone, the simple stress hormone (van Campen et al., 2018). Table 4 shows an overview of various biological markers associated with numerous physical, emotional and environmental stresses.

**Table 4:** Various biological markers associated with numerous physical, emotional and environmental stresses.

**S. No**.	**Biomarker molecule**	**Brief introduction**	**Efficacy of the molecule**	**Associated ailment(s)**	**Marker intended for**	**Diagnostic assay**	**Reference(s)**
1.	α-amylase	A salivary enzyme, estimated as the marker for sympathetic stimuli. In stress response the α-amylase concentration increases	Hydrolysis of internal α-1,4-glycosidic linkages in starch	Stress associated with periodontal disease	Physiologic and behavioral stress	Enzyme Immunoassay (EIA) kit	Rai and Kaur, 2011
2.	Chromogranin A (CgA)	A protein prohormone with acidic nature, present in the secretory granules of different neuroendocrine tissues	Influence endocrine, cardiovascular, and immune systems and glucose or calcium homeostasis	Indicative of endocrine tumors, cardiovascular, inflammatory, and neuropsychiatric diseases	Mental stress marker during monotonous driving	ELISA, immunoradiometric assay (IRMA) and RIA	Yamakoshi et al., 2009; D'amico et al., 2014; Gut et al., 2016; Mishra, 2017
3.	Secretory IgA	Present in external body secretions like saliva. The induction of stress hormones, especially cortisol, have an adverse impact on immunoglobulin secretion	Participate in immune function of mucous membrane	Immunosuppressive effects	Job stress	Immunofluorescence staining and flow-cytometry analysis	Lee et al., 2010
4.	MicroRNA-29c	Expressed in the human prefrontal cortex	Involved in psychopathologies, such as schizophrenia and bipolar disorder and Alzheimer's, Huntington's and Parkinson's diseases	Indicate stress-induced functional neural alterations	Social stress task	TaqMan Low Density array/TaqMan real-time PCR assay	Vaisvaser et al., 2016
5.	miR-16	Acute psychological stress-responsive miRNAs	Play a role in the inhibition of inflammatory cytokine response	Indicate positive correlation with anxiety	Chronic academic stress	miRNA microarray/TaqMan® MicroRNA assays	Honda et al., 2013
6.	miR-146a and−212 expression	Responsive to inflammatory stimuli	Deficiency result in an excessive IL-6 and TNF-α production, myeloproliferative syndrome, chronic inflammation and a decrease in the number and quality of hematopoietic stem cells	Postpartum psychosis	Postpartum psychotic stress	TaqMan array human microRNA A Cards v2.0	Weigelt et al., 2013; Testa et al., 2017
7.	Relative telomere length	Repetitive DNA sequences present at the ends of eukaryotic chromosomes that undergoes attrition after every somatic cell division	Shorter telomeres are indicative of accelerated aging	Fear disorders including generalized anxiety disorder (GAD), panic disorder, agoraphobia, and social and other phobia	High phobic anxiety in women	Real-time polymerase chain reaction	Okereke et al., 2012
8.	Sigma-1 receptor	An endoplasmic reticulum chaperone involved in regulation of bioenergetics, free radical generation, oxidative stress, unfolded protein response, cytokine signaling, morphogenesis of neuronal cells	Its ligands exhibit antidepressant-like and neuroprotective actions	Mental disorders	Psychological stress	Single-cell reverse transcription-polymerase chain reaction (scRT-PCR) and immunofluorescence staining	Hayashi, 2015; Zhang K. et al., 2017
9.	Biopyrrins (bilirubin oxidative metabolites)	Biosynthesized from heme	Considered as harmful and useless substance	Associated with the risk of bilirubin encephalopathy and neuronal injury	Psychological stress	ELISA	Miyaoka et al., 2005
10.	Glycated hemoglobin (HbA1C)	HbA1c is considered as a possible substitute to fasting blood glucose for diagnosis of diabetes.	Independent risk factor for coronary heart disease and stroke	Anxiety, irritability, mood swings, insomnia, depression, and a sense of failure	Physical and emotional exhaustion mediated stress	Hexokinase assay and turbidimetric immunoinhibition methods by automatic analyzer	Sherwani et al., 2016; Deneva et al., 2019
11.	Cardiac troponin I	Considered as gold-standard biomarker for detecting acute myocardial necrosis and acute myocardial infarction	Cardiac damage during HS recovery	Multi-organ failure	Heat stroke stress	Handheld iSTAT clinical analyzer	Quinn et al., 2014; Park et al., 2017
12.	Osteopontin	A phosphoglycoprotein marker of hypoxic conditions	Involved in biomineralization, immune regulation and inflammation	Acute mountain sickness, high-altitude cerebral edema, high-altitude pulmonary edema	High altitude stress	ELISA	Basnyat, 2018; Tang X. G. et al., 2018

HSPs are being accepted widely as potent biomarkers with multiple applications wherein lower HSP expressions have been linked to the manifestation of neurological disorders, cardiovascular disease, and cancer (Charmpilas et al., 2017). HSP90 and proinsulin are recognized as a stress biomarker of beta-cell stress in the early period of type 1 diabetes; HSP90 increases about four times in the islets from non-obese diabetics relative to those from controls (Watkins et al., 2016). Another study reported that salivary and circulatory HSP70 showed a significant increase in a renal dialysis patient, confirming that salivary and circulatory HSP70 is an efficient stress marker in chronic renal disease condition (Hegde and Nireeksha, 2016).

Prognostic biomarkers are getting much focus these days, owing to their importance in determining the outcome of a disease/condition as well as the therapy. Various APPs have also long been used as general prognostic and diagnostic biomarkers in a variety of injuries, independently of their location and cause. Proteomic techniques have demonstrated that APPs favor the systemic regulation of defense, coagulation, proteolysis, and tissue repair (Schrödl et al., 2016). Recently, the usefulness of biomarkers in cancer development, diagnosis, and prognostic accuracy has been reviewed by Dadar et al. (2016). DNA methylation has also been utilized as a biomarker for the prognosis and diagnosis of several cancers including breast, colon, liver, and lung (Hao et al., 2017). A glycoprotein having molecular mass of 40 kDa harboring three N-terminal amino acids namely tyrosine (Y), lysine (K), and leucine (L) known better as YKL-40 is considered as an ideal biomarker in serum for diseases indicated by inflammation, fibrosis, remodeling of the extracellular matrix and malignancy (Johansen, 2006). YKL-40 is proved to be a diagnostic marker with better sensitivity, specificity, and accuracy for detecting pleural effusion with malignancy (Shahanaze et al., 2018) and breast cancer prognosis in human subjects (Wan et al., 2017).

Promising results are observed in recent literature regarding the prospects of circulating pro-angiogenic miRNAs, such as miR-17-5p, miR-18a, miR-19b-1, miR-20a, miR-210, miR-296, and let-7f as novel biomarkers for predicting the risk and severity of gastric malignancy in human subjects (Peng W. et al., 2018). Scientists have developed a novel and cost-effective solid phase extraction process using magnetic β-cyclodextrin for the extraction and enrichment of the potential markers of gastric tumor, such as *p*-hydroxybenzoic acid and *p*-cresol which is eliminated in traces in the urine of human gastric tumor patients (Shi et al., 2019). Use of circulating miRNA in diagnostics of brain tumor is promising since other protein biomarkers, such as glial fibrillary acidic protein (GFAP) are restricted to CSF or other brain tissue. From the meta-analysis of different studies conducted in the last years, it can be concluded that six mi RNAs, such as miR-9, miR-15a, miR-16, miR-21, miR-23a, and miR-124 have better accuracy to consider as circulating biomarker for gliomas (Santangelo et al., 2017) Different serum exosomal miRNAs including miR-638 could be utilized for diagnosis and prognosis of hepatocellular carcinoma (Shi et al., 2018; Xue et al., 2019).

Another emerging candidate for clinical biomarker is the exosomes, which are the secretory vesicles released from cells and contain multiple compounds, such as lipids, proteins, RNAs, etc. (Théry et al., 2002). These vesicles were initially regarded as garbage bags of cells but later after intense exploration, were found to have critical roles in cell communication, physiological events as well as pathological changes (Bang and Thum, 2012; Sun et al., 2013). The findings on exosomes that they are released by multiple cell types and present in almost all body fluids, paved the way for identifying exosomes specific to cells of origin as novel biomarkers for clinical conditions also (Lin et al., 2015). It is the miRNAs present in exosomes that are largely evaluated for diagnosis of various conditions like cancer since exosomal miRNAs are resistant to RNase-mediated degradation (Hunter et al., 2008). In human ovarian cancer, eight miRNAs from the biopsy specimen which are previously recognized as specific biomarkers for the condition could be detected from the serum exosomes also, suggesting the scope of developing these exosomal miRNAs as surrogate diagnostic candidates as well as for biopsy profiling (Taylor and Gercel-Taylor, 2008). Another report on esophageal squamous cell carcinoma indicated the specificity of exosomal miR-21 as biomarker from serum which is detected in patients with benign tumor without any systemic inflammation and its level in serum was proportionate to the tumor progression, suggesting its role in prognosis also (Tanaka et al., 2013; Lin et al., 2015). Besides cancer, exosomal miRNAs were identified in predicting cardiovascular conditions, metabolic disorders, and renal dysfunctions (Hong et al., 2009; Kuwabara et al., 2011).

Most of the physiological markers are late to reflect their serum or urine levels in chronic kidney patients, so it is a herculean task to identify a urinary biomarker in patients suffering from chronic kidney disease at an early stage. Urinary exosomes are gaining importance among researchers as a potential bioactive fluid which can be targeted as a source of biomarkers in patients suffering from chronic kidney disease. In a recent study, it has been found that 30 non-coding (nc) RNAs are differentially expressed in the urinary exosomes at an early stage of kidney disease. Out of these, miRNA-181a is the most promising as it is shown a 200-fold difference in comparison to healthy subjects (Khurana et al., 2017).

Studies on neurological conditions like Alzheimers disease reported the involvement of exosomes in secreting out the disease-related β-amyloid peptides (Rajendran et al., 2006) as well as the phosphorylation of exosome-associated tau during early stages of the condition (Saman et al., 2012), thereby indicating the critical role of exosomes in cerebrospinal fluid as biological signatures. The ultimate product of metabolism of purine, i.e., urate has been identified in the recent past as an influential neuroprotectant to treat Parkinson's disease (PD) apart from being an important biomarker/predictor of the PD (Chen et al., 2012).

**Figure 7:**
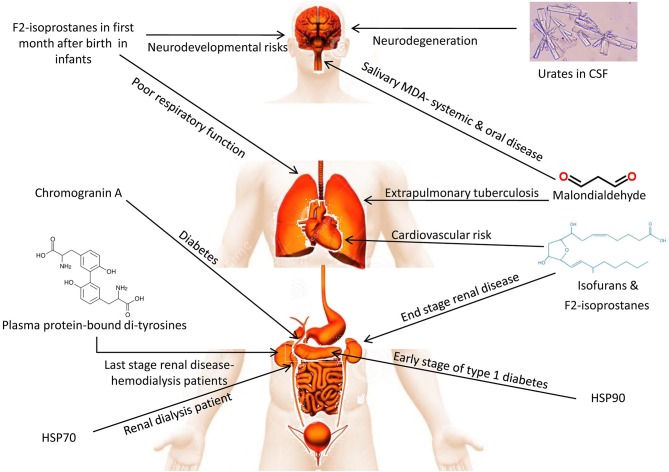
Biomarkers in prognosis of stress related diseases/disorders.

Identification and validation of disease-associated biomarkers play a crucial role in detecting diseases before their symptoms are observed, as well as in monitoring the outcome of therapies. Much focus is needed in designing point of care diagnostics based on biomarkers which need detailed and specific molecular profiling and specific screening using enough sample/population sizes. In conclusion, the important roles of stress biomarkers, their prognostic factors, and drug target properties represent a significant resource for future clinical applications and therapeutic approaches. Biomarkers in the prognosis of stress-related diseases/disorders are summarized in Figure 7.

## Clinical Significance of Biomarkers in Stress-Related Diseases—Highlights

Engineering robust biomarkers, either prognostic or predictive, is imperative for the advancement of clinical-related treatment strategies. Several successful research efforts have been made or being made to identify the relative usefulness of various biomarkers, including HSPs and APPs either in their pristine or combinational way in discriminating various stress-related diseases and disorders. The key scientific advances have revealed the uniqueness of biomarkers with special reference to the clinical arena. However, the prognostic or predictive values of the biomarkers mentioned above could not be ascertained in a statistically significant way and also to highlight the clinical manifestation, in-depth futuristic studies with larger clinical subjects may be warranted.

- Stress is an inevitable response in all organisms at the molecular to the whole-body level to maintain their homeostasis.
- Potential stress biomarkers act as molecular signatures found useful as non-invasive biological approaches for diagnosis, prognosis and treatment guidance.
- Diagnostic, prognostic and therapeutic values of valuable biomarkers have been highlighted in stress-mediated diseases and disorders.
- Various biomarkers have been proven as effective tools to diagnose the stress status as well as pathophysiological aspects of human/animal subjects.
- Identifying highly reliable biomarker is critical because the most promising markers need to be correlated with the particular conditions to facilitate prognosis and specificity of the therapy.

Some of the potent biomarkers which can be explored clinically are:

- Plasma and serum levels of malondialdehyde (MDA), isoprostanes, glutathione (GSH), and ROS reduction catalyzing enzymes, such as superoxide dismutase, catalase, myeloperoxidase, glutathione peroxidase, and thioredoxin reductase.
- Urate level as a promising biomarker to assess the incidence, diagnosis, and therapeutic prognosis of various neurodegenerative, hepatic, and renal diseases.
- APPs as indicators for social stresses, such as transportation, mixing, and abrupt weaning, which elicit an acute phase response.
- Sensitive neoplastic biomarkers can aid in the early detection and prognosis of neoplastic changes.
- Advances in molecular medicine have identified cell-free nucleic acids, including DNA, mRNA, and miRNAs as novel diagnostic markers for myopathies, recurrent exertional rhabdomyolysis, and osteochondrosis.
- Biocompounds, such as peroxilipids, malondialdehyde, etc. have been suggested to aid in disease diagnosis during early stages of gestation.
- HSPs are being accepted widely as potent biomarkers with multiple applications wherein lower HSP expressions have been linked to the manifestation of neurological disorders, cardiovascular disease, and cancer.
- Potent biomarkers for sensitizing renal injury include albumin, N-acetyl-β-D-glucosaminidase, kidney injury molecule-1, and exosomal transcription factors.
- Potent mediators of cardiovascular, CNS, hepatic, and nephrological disorders could be involved in the prognosis and treatment strategies.
- Stress biomarker research identified that oxidative biomarkers could be potential approaches for enantioselective toxicity control of pesticides.
- Non-invasive biological sources, such as saliva, urine, sweat, etc. provide optimum sources for the quantitative and qualitative assessment of chemical and physiological mediators associated with various conditions, such as stresses, diseases, and injury.
- Presently, quantitation of biomarkers is mostly based on immunological, chromatographical, and mass spectrophotometric assessments.
- Potent biomarkers which are efficiently validated can be employed in various reliable commercial assay kits.
- Advances in clinical techniques to assess various vital stages, as well as their underlying processes, will help to unravel the roles played by much more as-yet-undiscovered potent biomarkers, which ultimately will benefit the treatment and outcomes of patients.
- Further scientific and technical advances are yet to be explored, particularly regarding proteomic approaches, providing a wider chance to identify optimal and specific markers in any physiological/pathological disorder.

## Conclusion and Future Perspectives

In humans and animals, homeostasis is influenced highly by the physiological disturbances and molecular instabilities inflicted by various stressors, ultimately affecting their productivity, psychological health, and social welfare. According to the globally accepted one-health concept, where human health is highly intertwined with that of domestic and wild animals, there is a high demand to identify stress markers in the human and veterinary medicine sectors. There may be various biomarkers for each type of stress; however, identifying the best and most reliable biomarker(s) is critical, because the most promising markers need to be highly correlated with the specific pathophysiological aspects of the particular stress. They may include proteins, enzymes, hormones, chemicals, metabolites, genes, or by-products. Notable ones are malondialdehyde, isoprostanes, enzymatic antioxidants, blood urates, cortisol, copeptin, alpha-amylase, secretory IgA, chromogranin A (CgA), lysozyme, microRNAs (miRNAs), long non-coding RNAs (lnRNAs), heat shock proteins (HSPs), and acute phase proteins. Potent markers identified so far have been proven as effective tools to diagnose the stress status and have been applied to assess the prognosis and specificity of the therapy.

Diverse data from nature related to the identification of various types of physiological stresses and their associated molecular and systemic mediators, are still accumulating. Other novel technologies also could enable the development of non-molecular, functional, or biophysical tissue-based biomarkers. Moreover, the impact of sex differences and associated sex hormones over the expression of certain biomarkers need to be addressed. Therefore, the convergence of these biological, ecological, epidemiological, and zoonotic data, will lead their scientific scrutiny to identify promising markers to assess disease risk, diagnosis, prognosis, and therapeutic efficacy of drugs. Advances in clinical techniques to assess various vital stages, as well as their underlying processes, will help to unravel the roles played by many more as-yet-undiscovered potent biomarkers, which ultimately will benefit the treatment and outcomes of patients.

Stress is an inevitable culmination of all mammalian metabolism, which will be regulated normally within the physiological limits may skew exorbitantly following certain disturbances or diseases. This skewing or stress response may upshoot the concentration of different biochemical compounds within the cells or body fluids which could be potentially exploited as biomarkers for diagnosis, prognosis, and for therapy selection. Subcellular modification prevails during the stress response and disease progress –at the microarchitecture of the cell could facilitate positive clinical interpretations and remedial strategies. But the major deterrent in the practical use of stress biomarkers are uncertainty as to whether the reported response is a steady-state adaptation stage or the initial phase of a stress response. On the other hand, comparisons between experimental variants, plots, or species should only be made since an equal steady-state is marked. Another hitch in the exploitation of biomarkers clinically is the uncertainty that whether these are part of the stress-related diseases and disorders, and anti-oxidative defense systems or not. As the most of the known stress biomarkers as of date, such as thermal stress markers like heat shock proteins (HSPs), innate immune markers like acute phase proteins (APPs), oxidative stress markers, and chemical secretions in the saliva and urine are the part of anti-oxidant defense system of the body, a highly sensitive cut off value to differentiate their levels in different biological samples at different stages of stress or disease with respect to healthy condition is warranted. According to the results reported in this review, it is significant to involve multiple parameters, possibly covering potent mediators of cardiovascular, central nervous system, hepatic, and nephrological disorders, energy dissipation, and anti-oxidative defense, in human and animal-stress physiological studies. For example, in studies on animal tissues there is, at present, an attempt to measure as many systems related to cascade influences of vital early responding (e.g., cardiovascular, CNS, renal) and late responding (e.g., hepato-biliary, pancreatic) systems as possible.

Promising attempts to use several components of anti-oxidative and protective defense systems in human and animal tissues revealed different statistical approaches, which were profitably used to reveal typical arrangements of stress biomarker responses of animal and human at field plots. The first attempt toward the combination of such data proposes the metabolic modeling studies, which potentially gives perspective about multisystem involvement and hence widespread disturbance in a range of biomarkers through protective and stress pathways. From this perspective, the accurate evaluation of stress markers in saliva, urine, tears, and feces will turn to a critical part in measuring of the stress response of human and animal, although it is necessary to include a wide range of stress–physiological parameters.

Identification of many more useful markers is warranted because their clinical application as universal biomarkers demands the following properties:

1. The ease of collecting and processing the related biological specimen.
2. Stability and durability of the marker throughout the storage and evaluation period.
3. The availability of assays with sufficient specificity and sensitivity for the particular marker.

Moreover, identifying non-invasive methods to assess biomarkers has the potential to provide accurate data related to exercise-induced physiological and psychological stress. Furthermore, multiple stress biomarkers are detectable in ambulatory individuals and add prognostic value to standard risk factors for predicting death. So far, considerable advancements have been achieved in identifying and standardizing biomarkers of various origins. However, salivary and renal/urinary biomarkers are proving quite convenient. Present quantitation mostly based on immunological, chromatographical, and mass spectrophotometric assessments, have produced reliable results. However, some variability, and low reproducibility are also reported. Further scientific and technical advances are necessary, particularly regarding proteomic approaches, so that profiling of the markers in each biological sample becomes possible, providing a wider chance to identify optimal and specific markers in any physiological/pathological disorder. Hopefully, future advances will provide us with precise, multidimensional biological molecules that can address many of the current diagnostic and therapeutic deficiencies.

## Author Contributions

KD, SL, MD, HS, and RT initiated this review compilation. RK, RT, SC, MY, PB, and KK updated the current progress of identifying biomarkers. RK designed the table. AM and KK designed the figures. KD, HI, AM, SJ, KS, and WC reviewed, analyzed, and edited the final version. All the authors substantially contributed to the conception, design, analysis, interpretation of data, checking and approving the final version of the manuscript, and agree to be accountable for its contents.

### Conflict of Interest

The authors declare that the research was conducted in the absence of any commercial or financial relationships that could be construed as a potential conflict of interest.

## Abbreviations

AKI: acute kidney injury
APPs: acute phase proteins
Adipo-IR: adipose tissue insulin resistance index
ACTH: adrenocorticotropic hormone
AGEs: advanced glycation end products
AOPP: advanced oxidation protein products
ALAT: alanine-aminotransferase
ALP: alkaline phosphatase
AD: Alzheimer's disease
ALS: amyotrophic lateral sclerosis
ASAT: aspartate-aminotransferase
Asp: aspartic acid
ADHD: Attention-Deficit/Hyperactivity Disorder
AIx: augmentation index
ASDs: autism spectrum disorders
Bcl-2: B-cell lymphoma 2
Bcl-xL: B-cell lymphoma extralarge
BP: blood pressure
BUN: blood urea nitrogen
BNP: brain-type natriuretic peptide
CCL2: CC-chemokine ligand 2
CNS: central nervous system
CgA: chromogranin A
COPD: chronic obstructive pulmonary disease
CRH: corticotropin-releasing hormone
CRP: C-reactive proteins
DAXX: death domain associated protein
DN: diabetic nephropathy
DG: diacylglycerols
ELISAs: enzyme-linked immunosorbent assays
eGFR: estimated glomerular filtration rate
EBC: exhaled breath condensate
ETFs: exosomal transcription factors
F2-Isops: F2-isoprostane
FMD: Flow Mediated Dilation
FFA: free fatty acid
GC/MS/MS: gas chromatography coupled with tandem mass spectrometry
GIP: gastric inhibitory polypeptide
GLP-1: glucagon-like peptide-1
GRP78: glucose-related protein 78
Glu: glutamic acid
GSH: glutathione
GSSG: glutathione disulfide
GPX: glutathione peroxidase
HF: heart failure
HSPs: heat shock proteins
HbA1c: hemoglobin A1c
HEL: hexanoyl-lysine
HPLC: high performance liquid chromatography
HDL: high-density lipoprotein
HPLC-DAD: HPLC with diode-array detection
HODE: hydroxyoctadecadienoic acid
HPA: hypothalamic-pituitary-adrenocortical
IGFBP7: insulin-like growth factor-binding protein 7
IL-1RA: interleukin 1 receptor antagonist
IL-22: Interleukin-22
LPO: lipid peroxidation
LBP: lipopolysaccharide-binding protein
LDL: lipoprotein
liver IR: liver insulin resistance
liver IS index: liver somatic index
L-FABP: liver-type fatty acid-binding protein
LDL: low-density lipoprotein
MRI: magnetic resonance imaging
MAP: major acute phase protein
MDA: malondialdehyde
MDA-LDL: malondialdehyde-modified low-density lipoprotein
Met: methionine
miRNAs: microRNAs
MG: monoacylglycerol
MCP-1: monocyte chemoattractant protein-1
MCL1: myeloid cell leukemia sequence 1
MPO: myeloperoxidase
PUFAs: N-3 polyunsaturated fatty acids
NO: nitric oxide
NEFA: non-esterified fatty acids
Orn: Ornithine
oxLDL: oxidized low density lipoprotein
HOMA-B: pancreatic B-cell function
PD: Parkinson's disease
PHE: phenylalanine
PTSD: post-traumatic stress disorder
PTSD: post-traumatic stress disorder
RNS: reactive nitrogen species
ROS: reactive oxygen species
RSI: reactive species interactome
RBCs: red blood cells
Tregs: regulatory T lymphocytes
SAA: serum amyloid A
SAP: serum amyloid P
Ask 1: signal-regulating kinase 1
sICAM: soluble intercellular adhesion molecule
SCD: stearoyl-CoA-desaturase
SOD: superoxide dismutase
SOCS: suppressor of cytokine signaling
SAM: sympathetic adreno-medullary
TBARS: thiobarbituric acid-reactive substances
TAS: total anti-oxidant status
TAS: total anti-oxidant status
HNE: trans-4-hydroxy-2-nonenal
TRP: transient receptor potential
TG: Triglyceride
Tyr: Tyrosine
UA-LLsME: ultrasound-assisted liquid-liquid semi-microextraction
uHSP72: urinary heat shock protein 72
sVCAM: vascular cell adhesion molecule-1
AGP: α1-acid glycoprotein
BD2: β-defensin 2
GGT: γ-glutamyltransferase
4-HNE: 4-hydroxy-2-nonenal
8-OH-dG: 8-hydroxydeoxyguanosine
8-iso-PGF2α: 8-iso-prostaglandin F2α
8-ISO: 8-isoprostane
NF-κB: nuclear factor-kappa B.
